# Communication strategies for delivering personalised dementia care and support: a mixed-methods systematic review and narrative synthesis

**DOI:** 10.1093/ageing/afaf120

**Published:** 2025-05-15

**Authors:** Astrid Maria Sunjaya, Tamar Schreiber, Kumud Kantilal, Nathan Davies, Sarah Griffiths

**Affiliations:** University College London, Research Department of Epidemiology and Public Health, London, UK; UCL Medical School, University College London, London, UK; University College London, Research Department of Primary Care and Population Health, London, UK; Queen Mary University of London, Wolfson Institute of Population Health, Centre for Psychiatry and Mental Health, London, UK; University College London, Research Department of Primary Care and Population Health, London, UK

**Keywords:** dementia, healthcare, personalised care, communication, systematic review, older people

## Abstract

**Background:**

Given the significant burden and rising prevalence of dementia, it is essential that personalised care is available to people with dementia (PWD) and their family carers. This involves tailoring support to meet individuals’ unique needs and preferences. Effective communication is fundamental to delivering such care, yet dementia impacts communication, posing challenges in meeting individuals’ needs.

**Aim:**

To understand key communication strategies used by healthcare professionals (HCPs) in delivering personalised dementia care.

**Methods:**

A systematic search across MEDLINE, EMBASE, EMCare, PsycINFO, CINAHL, Scopus, and Web of Science was conducted (April 2024) without limits on care setting, country or publication date. We identified studies examining communication strategies, barriers, and facilitators for delivering personalised care for PWD and their carers. Study quality was assessed using Joanna Briggs Institute critical appraisal tools and the Mixed Methods Appraisal Tool. Using codebook thematic analysis, a narrative synthesis of findings was developed.

**Results and Conclusion:**

The review included 33 studies, encompassing qualitative, quantitative and mixed-methods research conducted in hospitals, care homes and community settings. Most studies originate from high-income countries and care homes, limiting generalisability. Three themes on communication strategies for delivering personalised dementia care were developed: understanding the person, their family and their care context; communication techniques (verbal, nonverbal and use of external aids); and support for the workforce. The review underscores the importance of combining practical, emotional and relational approaches while highlighting current gaps, such as the need for better workforce support and more research in primary care and culturally diverse contexts.

## Key Points

- Understanding the person was a central pillar of communication when delivering personalised dementia care.
- Effective personalised dementia care relies on combining practical with emotional and relational communication strategies.
- Gaps include the need for better workforce support, and more research in primary care and culturally diverse contexts.

## Introduction

Dementia, characterised by cognitive decline impacting memory, thinking and the performance of daily activities [1], affects 55 million people worldwide [2], with projections reaching 131.5 million by 2050 [3]. The economic [3] and social [4] burden is significant, with increasing pressures on health and social care [5]. Navigating postdiagnostic support for people with dementia (PWD) is difficult with services being complex to navigate, limited or unavailable [6].

Person-centred care is widely recognised as a priority in national dementia guidelines [7]. As described by Kitwood, this involves treating PWD with dignity and respect, acknowledging their personal history and needs and focusing on maintaining identity and wellbeing through compassion [8]. Informed by Kitwood, Brooker developed the VIPS framework to guide comprehensive care [9]. This takes account of a person’s values (V), provides individualised (I) care according to needs and understands care from the perspective (P) of the person with dementia, within a social environment (S), allowing supportive relationships. NICE guidelines highlight the importance of valuing the individuality of PWD and their relationships and addressing carer needs [10].

Personalised care, built on person-centred care principles, aims to provide support tailored to individual needs [11]. The World Alzheimer’s Report 2022 advocates for personalised care plans, enabling PWD and their carers to make informed decisions and plan for the future [6]. Similarly, National Health Service (NHS) England recommends a personalised care and support plan, updated annually and developed through proactive communication between healthcare professionals (HCPs) and PWD [12].

Effective communication is essential for personalised care, allowing HCPs to understand people’s unique needs and preferences [13, 14]. However, communication challenges frequently associated with dementia, including reduced comprehension, reduced verbal expression and memory problems, complicate these conversations [15–17]. Cultural and language diversity adds complexity, necessitating culturally competent communication and care [18, 19]. Despite this, there is little evidence-based guidance for HCPs on facilitating personalised care conversations. NHS England’s guidance on Good Personalised Care and Support Planning in dementia advises on domains of care to cover, and multidisciplinary involvement, but lacks specific guidance on how to have such conversations [12]. Existing evidence reviews on dementia-related communication strategies have focused on specific settings, for instance, care homes [20], outpatient clinics [21] and internet-based guidelines and advice [22], or specific professions, such as nursing [23]. To our knowledge, despite the significance of interaction for personalised care delivery, there are no reviews incorporating the concept of personalised care and how this can be achieved through communication strategies, across a range of healthcare settings and practitioners.

This review aims to understand key communication strategies and concepts used by HCPs in delivering personalised dementia care across a range of healthcare settings. The review will inform personalised dementia care delivery and ultimately support the quality and consistency of dementia care.

## Methods

This systematic review was conducted and reported following the Preferred Reporting Items for Systematic Reviews and Meta-Analyses Protocols (PRISMA-P) guidelines [24]. The systematic review protocol was published in PROSPERO (ID CRD42024527933).

### Search strategy and selection criteria

A search was conducted in April 2024 across the following databases: MEDLINE, EMBASE, EMCare, PsycINFO, CINAHL, Scopus and Web of Science. A citation search was also carried out. Complete search terms are listed in the supplementary file, Appendix 1. No limits were placed on date, country or language.

Search results were deduplicated in EndNote [25] and screened in Rayyan [26]. Two reviewers independently screened the studies, first by title and abstract and then by full text. A.S. screened all studies, while T.S. screened 20%. Discrepancies were resolved through discussion with K.K. and S.G. This process was presented through a PRISMA flowchart.

Eligibility criteria were designed using the SPIDER (Sample, Phenomenon of Interest, Design, Evaluation, Research type) framework [27], outlined in Table 1.

**Table 1:** Eligibility criteria.

	Inclusion criteria	Exclusion criteria
**S: Sample**	PWD and/or their carers as the focus.	– Sample excludes PWD and/or their carers. – Mixed sample (e.g. only a proportion have dementia or are carers of PWD).
**P: Phenomenon of interest**	Studies addressing at least one broad domain of personalised care and support^a^ for PWD and their carers, in combination with communication barriers to and/or strategies for identifying and/or addressing (delivering) these. This includes studies focused on Advance Care Planning (ACP).	– Studies about dementia screening and diagnosis that focus solely on presenting a diagnosis (we included studies on communication designed to support understanding of diagnoses and their impact). – Studies strictly focused on the interaction between PWD and informal carers. – Studies where the focus is on therapy or intervention for a dementia symptom. – Studies where the focus is entirely on implementation strategies for a model of person-centred care.
**D: Design**	No limits on setting: include primary/community/secondary and care home settings.	
**R: Research type**	– Quantitative studies – Qualitative studies – Mixed-methods studies	– Literature reviews – Meeting abstracts – Commentary, opinion and theoretical pieces

^a^i.e. Services involved, information needs, social and personal history, home/care environment, activities of daily living, activities and interests, impact of psychosocial and cultural factors, impact of diagnosis, cognitive and behavioural changes, planning for contingencies and changes, progression and end-of-life care, safeguarding and advocacy and physical health and medication management [12, 28].

### Quality assessment

Quality of included qualitative and quantitative studies was assessed using the Joanna Briggs Institute (JBI) Critical Appraisal Tools [29]. Mixed-method studies were assessed using the Mixed Methods Appraisal Tool (MMAT) [30]. Studies were not excluded based on quality assessment, but the quality was always reported and used to describe studies and weight the evidence. Appraisal was conducted independently by A.S. and T.S. Disagreements were resolved by K.K. and S.G.

### Data extraction

Data from included studies were extracted using a Microsoft Excel template and included: title, author, year of publication, aim, study design, country of study, context of study, participants’ demographics, sample size, severity of dementia, details of the communication strategy and main findings. Data extraction was completed by A.S., with T.S. checking 20% of the studies.

### Data synthesis

A narrative synthesis was conducted following Popay et al.’s four-stage approach: developing a theory of how the intervention works, why and for whom; developing a preliminary synthesis of the findings from the included studies; exploring relationships in the data; and assessing the robustness of the synthesis [31].

A codebook thematic analysis [32] was used to synthesise findings and explore relationships in the data, carried out by A.S. and S.G. For the initial coding exercise, we selected 6/33 papers (~20%) that reflected a range of methodologies and settings. Initial codes were generated inductively for phenomena relating to the research question, to develop a preliminary codebook, which was then applied deductively and inductively across the full data set [33, 34]. The codebook was iteratively refined throughout this whole process, adding and refining codes throughout. Based on team discussions, codes were organised into groups by identifying patterns and relationships between them and then developed into themes. A table and a concept map were created to summarise themes and subthemes. Robustness of the synthesis was assessed by considering the quality and heterogeneity of the studies.

## Results

### Study inclusion

**Figure 1:**
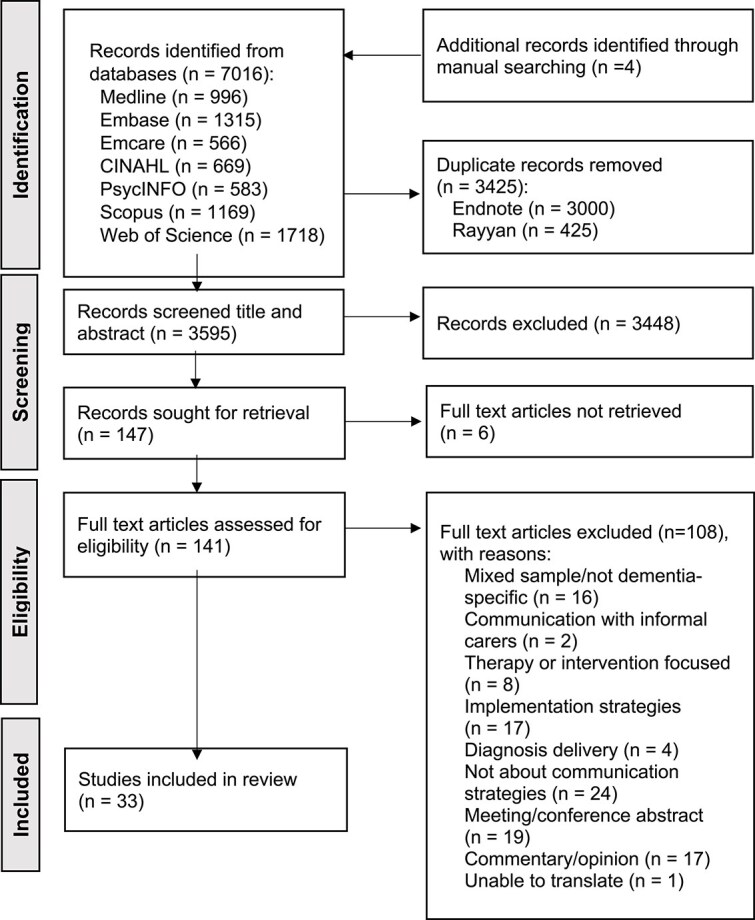
PRISMA Flowchart [24].

The database searches yielded 3591 unique studies, with four additional records identified via citation searching, and 147 studies screened by full text. Of these, 33 studies were included in the final review. Details of search results are documented in the PRISMA flowchart (Figure 1).

### Description of the studies

The 33 included studies were conducted primarily in high-income countries. Of these, 15 originated from the USA [35–49]; 9 from the UK [50–58]; 4 from Canada [59–62]; 2 from Portugal [63, 64]; and 1 each from Spain [65], Taiwan [66] and Norway [67]. Twenty-one studies were conducted in care homes [35, 37–45, 48, 49, 56, 58, 59, 62–67], three in home care settings [55, 60, 61], two in hospital outpatient settings [47, 54], four in hospital inpatient settings [46, 50–52], one in a community-based setting [53] and two in mixed settings [36, 57].

Observational studies include 7 cross-sectional, 12 qualitative and 2 mixed-methods studies. These studies observed communication patterns between HCPs and PWD and perspectives on barriers and facilitators of effective communication. There were 10 quasi-experimental studies and 2 cluster randomised controlled trials (RCTs), which evaluated the effectiveness of specific communication strategies with PWD or evaluated the impact of staff training on communication techniques. Key characteristics of the included studies are summarised in Table 2, with detailed data extraction available in the supplementary file, Appendix 2.

**Table 2:** Summary of included studies.

Author (year)	Country, setting^*^	Aim	Design	Dementia severity	Participants	Communication strategies	Main findings
Acton et al. (2007) [35]	USA, CH	To test the effect of individualised communication prescriptions on social communication between PWD and nurse.	Quasi-experimental	Mild, moderate, severe	10 residents	Avoid open leads, use focused leads, allow extra time for response, use minimal cues, avoid yes/no and specific recall questions.	Increase in participant’s words per topic and percentage of topics introduced. Participants with lower MMSE scores showed the most improvement.
Allwood et al. (2017) [50]	UK, HI	To examine HCPs’ close encounters with PWD in acute settings and identify barriers to effective closing.	Qualitative	NR^**^	26 patients, 9 doctors, 11 nurses, 6 allied health professionals	Closing encounters with open-ended preclosings, mixed messages, nonspecific terms.	Open-ended questions led to difficulties and mixed messages caused confusion and resistance. Concrete arrangements preferred.
Alnes et al. (2011) [67]	Norway, CH	To investigate changes in interactions between PWD and nurses during morning care post-Marte Meo Counselling (MMC).	Quasi-experimental	Moderate to severe	10 residents, 13 nurses	Function-Supporting Elements (FSE): Prepare for positive beginnings, locate and follow focus, state what is happening and what is going to happen, reinforce coping ability, wait for answers and pay attention to physical contact. Inappropriate Interaction (II): inaccurate, unnecessary information, memory challenge, commanding tone, inattentive.	Intervention group was stable or scored higher on FSEs after MMC. II was more pronounced in the control group. MMC improved awareness of residents’ capabilities, increasing self-confidence in caregiving roles.
Anantapong et al. (2022) [51]	UK, HI	To understand the experiences, views and needs of family carers and hospital staff regarding communication about nutrition and hydration in severe dementia.	Qualitative	Severe	12 family carers, 17 staff	Early discussions with PWD and family members, multidisciplinary involvement, clear steps ahead, validation, empathic reassurance, be sensitive to emotions and values.	Themes developed from interviews: prerequisites to initiating communication about eating and drinking, communication aiming to develop agreed care plans, difficulty discussing palliative and end-of-life care, needs of information and plans about future eating and drinking difficulties.
Baillie et al. (2012) [52]	UK, HI	To explore nursing students’ experiences in caring for PWD in acute settings.	Qualitative	NR	Four focus groups, four to six students in each group	Getting to know the person and building relationships, involvement of families, flexible care, providing comfort and reassurance.	Communication strategies aligned with person-centred care. Students often had to negotiate these approaches with hospital routines.
Barbosa et al. (2016) [63]	Portugal, CH	To assess the effects of a psycho-educational intervention on care workers’ communicative behaviours with residents during morning care.	Quasi-experimental	Moderate to severe	47 residents, 58 care workers	Communicative strategies (e.g. give simple choices, use validation, allow time to respond, use individual’s name and eye contact), enhance environment, motor and multisensory stimulation.	Psycho-educational intervention had a broader positive impact compared to education-only intervention. Some positive behaviour dropped at 6-month follow-up.
Barbosa et al. (2016) [64]		To examine the 6-month effects of the above study					
Benbow et al. (2011) [53]	UK, C	To identify the skills PWD and carers felt were needed in the dementia care workforce.	Qualitative	NR	11 PWD, 11 carers’ group members, 47 dementia cafe attenders	Knowledge about dementia; person-centred care; communication about fears, hopes and challenges; building relationships; support for carers and helping people engage in activities	Skills needed identified in the previous column.
Berry et al. (2023) [36]	USA, M	To adapt the Serious Illness Conversation Guide (SICG) for ACP conversation with PWD.	Mixed method	NR	14 patients, 18 caregivers	Alteration to SICG: use dementia-specific language, caregiver-directed support.	The adapted guide facilitated effective triadic communication between patients, caregivers and clinicians.
Bourgeois et al. (2001) [37]	USA, CH	To examine the effect of memory aids on conversations between nursing aides and residents.	Cluster RCT	Moderate	66 residents, 66 nursing aides	Memory books used as cues during care activities to facilitate cooperation and as distraction tools.	Increased verbal interactions and informative utterances when memory aids were used. Nursing aides’ perception of residents’ depression improved.
Bourgeois et al. (2004) [38]	USA, CH	To examine the effect of communication skills training for nursing aides.	Quasi-experimental	Moderate	125 residents, 126 nursing aides, 23 LPNs	Effective skills (announce care, address by name, wait for response), effective instructions (short and clear, positive feedback, use memory books).	Improvement in effective skills post-training, maintained at 3-month follow-up.
Burshnic and Bourgeois (2022) [39]	USA, CH	To examine the impact of using external support in response to preference questions.	Quasi-experimental	Severe	21 residents	Preference questions paired with photographs and simple text; responses facilitated through a sorting mat.	Resident’s comprehension improved with a supported assessment method.
Dooley et al. (2018) [54]	UK, HO	To examine how doctors involve PWD in medication decisions and the impact on patient acceptance.	Cross-sectional	Mild	71 patients with 67 companions, 21 doctors	Treatment recommendation: pronouncements, proposals, suggestions, offers, assertions.	Suggestions led to higher resistance. Pronouncements led to passive resistance. Satisfaction was lower with pronouncements.
Douglas and MacPherson (2021) [40]	USA, CH	To assess the impact of a coaching strategy on nursing assistants’ communication behaviours and PWD behaviours.	Quasi-experimental	NR	7 PWD, 7 Certified Nursing Assistants (CNAs)	Eye contact, smiling, greeting with name, external memory supports, guiding to activity.	Statistically significant increase in positive communication behaviours and decrease in negative responsive behaviours postcoaching.
Kaasalainen et al. (2021) [59]	Canada, CH	To evaluate the implementation of the Conversation Starter Kit (CSK).	Quasi-experimental	NR	55 residents, 35 family members	CSK booklet to facilitate ACP discussions.	Using the booklet results in higher engagement score but a decrease in the self-efficacy score of family members.
Kamalraj et al. (2021) [60]	Canada, CC	To explore formal caregivers’ experiences in communicating with PWD at home.	Qualitative	NR	15 Personal Support Workers (PSWs)	Positive (appropriate rate of speech) and negative (directive communication, threatening with repercussion) strategies, nonverbal communication (body language and attitude), home environmental cues.	Challenges due to dementia-related impairments and the emotional toll. Valuing communication in care. Home environment played a dual role, sometimes aiding and sometimes complicating communication.
Mundadan et al. (2023) [61]	Canada, CC	To investigate the overlap between language-based and person-centred communication (PCC) strategies in home care.	Cross-sectional	NR	12 residents, 11 PSWs	Language-based strategies, positive person work (recognition, negotiation, validation, facilitation).	Several language-based strategies support PCC goals.
Palmer (2012) [41]	USA, CH	To explore caregivers’ communication patterns and desired communication with nursing home staff.	Qualitative	NR	15 family caregivers	TALKKK: Tell, Ask, Listen, Know, Be Knowledgeable, Share Knowledge.	Acknowledging family caregivers as partners in care.
Passalacqua and Harwood (2012) [42]	USA, CH	To examine the feasibility of a VIPS-based communication intervention for caregivers.	Quasi-experimental	NR	26 facility caregivers	VIPS Model: Valuing people, individualised care, personal perspectives, social environment.	Communication intervention reduced depersonalisation and increased empathy. Resistance to reducing pet names was noted.
Riachi (2017) [55]	UK, CC	To investigate the use of SPECAL™ techniques in maintaining well-being in clients with dementia.	Qualitative	NR	5 care workers, 2 senior managers and trainers	Avoid questions, listen, do not contradict, protecting, reassurance, empathy.	SPECAL™ maintained client personhood and self-esteem. Regular supervision was crucial for effective use.
Saini et al. (2016) [56]	UK, CH	To examine and improve practices relating to end-of-life discussions with family members.	Qualitative	NR	4 family members, 19 HCPs	Educating family and staff, ongoing dialogue, in-depth discussions.	Education for families and confidence-building for staff are crucial. Cultural issues and task-oriented approaches were barriers.
Savundranayagam et al. (2014) [43]	USA, CH	To assess whether staff–resident interactions during caregiving tasks were person-centred.	Cross-sectional	Moderate to severe	13 staff–resident dyads	Recognition, negotiation, facilitation, validation.	Person-centred communication was possible during care tasks, but opportunities were often missed.
Savundranayagam and Moore-Nielsen (2015) [44]		To examine the overlap between language-based strategies and PCC indicators.	Cross-sectional			Language-based strategies, PCC: recognition, negotiation, facilitation, validation.	Overlaps were found between language-based strategies and PCC. Training needed to diversify language strategies.
Savundranayagam et al. (2016) [45]		To examine resident reactions to PCC during routine care tasks.	Cross-sectional			Recognition, negotiation, facilitation, validation.	Increased PCC led to more positive resident reactions. Missed opportunities led to negative reactions.
Shaw et al. (2022) [46]	USA, HI	To describe attributes of elderspeak use in hospital dementia care and determine associated characteristics.	Cross-sectional	Moderate to severe	16 patients, 53 staff	Elderspeak: infantilising words, directive phrases, mitigating expressions, exaggerated praise, tag questions, interruptions, laughing at or belittling, altered prosodic.	Elderspeak was prevalent in 96.6% of care encounters. Older staff used it more frequently. Nonsignificant factors included dementia severity.
Sánchez-Martínez et al. (2023) [65]	Spain, CH	To evaluate changes in communication of professionals after training.	Quasi-experimental	NR	8 nurse aides, 1 physiotherapist, 2 social educators	Nonverbal techniques (centring, empathy, observing), verbal (ambiguity, trusted listening, reminiscence, rephrasing).	Training improved professionals’ understanding of patients’ behaviours, changed the communication strategies and induced emotional reactions.
Song et al. (2019) [47]	USA, HO	To adapt SPIRIT intervention for PWD and assess participation in ACP.	Mixed methods	Mild to moderate	23 PWDs and their surrogates	SPIRIT: Assessing illness representation, identifying gaps and concerns, creating conditions for conceptual change, introducing replacement information, summarising, setting goals.	PWDs could articulate values and end-of-life wishes. Decision-making capacity may be more critical than global cognitive function.
Wang et al. (2013) [66]	Taiwan, CH	To explore communication difficulties faced by nurses interacting with PWD.	Qualitative	Moderate to severe	15 nurses	Language differences, blocked messages, difficulty interpreting emotions and needs.	Communication difficulties often leading to communication breakdowns.
Ward et al. (2008) [57]	UK, M	To explore communication patterns and the impact of the environment on dementia care.	Qualitative	NR	17 residents, 38 staff	Care speak: a distinct style and pattern of speech used by care workers when performing a task.	Care speak dominated communication; limits input by the resident.
Wheeler and Oyebode (2010) [58]	UK, CH	To gather care home staff’s views on communication issues and explore strategies to improve communication skills.	Qualitative	NR	36 direct care staff	Talking while doing, reminiscence, empowerment, disempowerment, reassurance, involving families and keeping them informed.	Person-centred approaches improved communication. Task-oriented approach leads to disempowerment. Conflicts with families may arise from different opinions on care.
Williams et al. (2009) [48]	USA, CH	To assess the impact of elderspeak communication on PWD’s response to care.	Cross-sectional	Moderate	20 residents, 52 nursing staff	Elderspeak: simplistic vocabulary and grammar, slowed speech, elevated pitch and volume, diminutives, collective pronouns, tag questions.	Elderspeak led to increased resistance to care.
Williams et al. (2017) [49]	USA, CH	To evaluate the effectiveness of a communication intervention in reducing resistiveness to care in dementia.	Cluster RCT	Moderate to severe	27 residents, 29 staff	Changing talk intervention to reduce elderspeak.	Elderspeak declined postintervention and at 3-month follow-up.
Wilson et al. (2012) [62]	Canada, CH	To identify effective communication strategies during ADLs for residents.	Qualitative	NR	10 formal caregivers	Task-focused communication strategies (verbal and nonverbal), social communication strategies (greeting, complimenting, responding), miscellaneous communication strategies (physical assistance, redirect). Emergent themes: be patient, focus on the resident, environmental cues, para-verbal monitoring, familiarity, assess mood, request assistance and postponed/repeated attempts.	Verbal strategies were rated more effective than nonverbal. Differences in strategies used based on dementia severity.

^a^Study setting: CH (Care Home), CC (Care in Community), HI (Hospital Inpatient), HO (Hospital Outpatient), C (Community), M (Mixed).

^b^NR (Not Reported)

### Quality assessment

The quality of studies was mixed, with some demonstrating strong methodological rigour while others had notable limitations. Qualitative studies generally posed clear research questions and employed appropriate methodologies; however, all 12 lacked explicit discussions on researcher reflexivity and positionality. Among cross-sectional studies, four failed to address confounding factors or describe their management, while one also did not clearly define inclusion criteria, study subjects or settings. Both RCTs lacked clear descriptions of allocation procedures, and blinding was not conducted due to the nature of the interventions. Additionally, one study had unclear randomisation procedures. Quasi-experimental studies were limited by the absence of control groups in six studies, small sample sizes in two studies and reliance on self-reported data in two studies. Supplementary file, Appendix 3 shows detailed quality assessment ratings.

### Review findings

**Figure 2:**
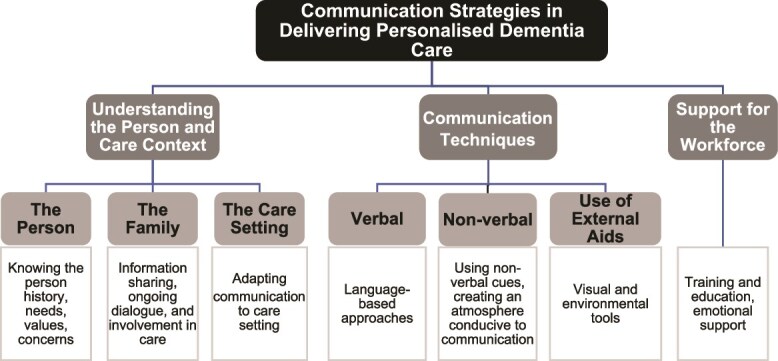
Concept map of communication strategies in personalised dementia care.

We developed three major themes in communication strategies for personalised dementia care: understanding the person and care context, communication techniques and workforce support. These themes are represented in Figure 2.

## Understanding the person and care context

### The person

#### Knowing their history

Studies highlighted the importance of understanding the personal history of PWD. Obtaining biographical details helps practitioners to incorporate life histories into conversation, support care planning and foster respectful relationships [43, 52, 58]. Familiarity with past life events and hobbies enables practitioners to identify what makes people happy and what might cause distress [42, 55]. A lack of historical knowledge can hinder a practitioner’s ability to detect and respond to PWD’s needs effectively [65]. Using reminiscence in conversation further enhances patient engagement and experience [58].

#### Knowing their ideas, values, needs and concerns

Understanding the ideas, values, needs and concerns of PWD is essential for personalised care. PWD and their carers express a strong need to discuss their fears, hopes and challenges [53]. Practitioners stress the importance of developing emotional connections with PWD by understanding their desires, habits and routines [41]. In acute settings, hospital staff must fully grasp the current situation of PWD before initiating care planning discussions, ensuring conversations are grounded in accurate and relevant information [51]. Effective negotiation and reaching agreements on care preferences depend on understanding these needs and preferences [45, 62]. Moreover, recognising and addressing people’s concerns in end-of-life conversations is vital to ensuring their values are respected [47, 56].

### The family

#### Information sharing and ongoing dialogue

Families can be key sources of information about PWD’s preferences, habits and history [52, 58]. They also seek updates from practitioners on their relative’s status, including care, medication and disease progression, to feel reassured and better equipped to provide support [41, 51]. Ongoing dialogue, rather than a one-time exchange, keeps families engaged and informed [56] through verbal knowledge-sharing sessions [58] or written information [56]. To ensure PWD and their carers are not marginalised and receive appropriate information at different stages, they also emphasise the need for practitioners to share their knowledge of dementia [53].

#### Family involvement in care

Practitioners have varied views on family involvement in dementia care. Family presence often supports communication but can create awkwardness for staff [60]. For instance, families rushing care routines can interfere with staff efforts to effectively communicate with the person [60]. Some relatives may be reluctant to engage in care planning, which ideally should involve conversations between people with dementia, staff and family members, due to satisfaction with current care, perceived irrelevance or emotional distance. Conflicts can arise from differing opinions on the best approach to care, which can derail care planning conversations [51, 58].

### The care setting

Adapting communication strategies to care settings is important. In settings such as hospital inpatient, care homes and home care, care task demands often lead to missed opportunities for meaningful communication [51, 52, 58, 60]. Care home staff face conflicting pressures between building relationships with residents and managing workloads, often relying on ‘care speak’, a directive, task-focused communication, restricting meaningful interaction and discouraging contributions from PWD [57]. In hospitals, barriers include the lack of opportunities for families to share important information and the need to repeatedly convey the same information to different staff members [51, 52].

However, integrating communication into routine tasks can create valuable opportunities for interaction [58]. Task-focused communication strategies, including using the person’s name to gain attention and negotiating based on their preferences, are identified as useful [62]. Enhancing the physical environment, by reducing background noise or providing private spaces for sensitive conversations, further supports communication [56, 63].

## Communication techniques

### Verbal communication techniques

#### Facilitators

Several verbal communication techniques have been identified as facilitators for enhancing person-centred care. Language-based strategies described by Savundranayagam [44] including greetings, rephrasing, using yes/no and open-ended questions and affirmations, were found to closely overlap with Kitwood’s person-centred communication principles: Recognition, Negotiation, Facilitation and Validation [44]. For example, yes/no questions facilitate Negotiation, while affirmations support Validation of a person’s feelings [44, 45, 61].

Addressing the person by name and using greetings helps establish a connection and foster respect [38, 40, 61–63]. Providing short, clear instructions [38, 52] and breaking down tasks into manageable steps can support comprehension and reduce anxiety and resistance [38, 61, 62, 67]. For example, ‘We’re going to brush your teeth now… I’m going to help you turn the water on now… Ms. X, here is the toothbrush’ [62]. Positive reinforcement, such as giving praise and positive feedback when PWD follow directions, encourages participation [38, 62], while repeated positive messages can provide reassurance and reduce stress [55].

Successful communication also involves avoiding contradictions and using techniques like saying ‘silly me’ to handle disagreements without escalating stress, followed by a statement that confirms the person’s view [55]. Indirect repair, where a listener repeats or rephrases a statement to maintain conversational flow without correcting the PWD, is effective in keeping communication smooth and nonconfrontational [42]. For instance, instead of correcting someone who says, ‘We’re in Spain right now’, a response like ‘This is Spain?’ maintains conversation and avoids disorientation [42].

Using focused leads, like conversational cues, maintains clarity and direction, aiding PWD to engage meaningfully, e.g. ‘We certainly have had interesting weather lately, haven’t we?’ [35]. Communication techniques like rephrasing, verbatim repetition and paraphrased repetition reinforce understanding and aid comprehension [62]. However, ambiguity in language, such as nonspecific pronouns (that, something), can be useful when practitioners do not fully understand phrases used by PWD, allowing continued conversation [65].

Finally, clear communication is important in concluding interactions. This involves making specific arrangements for what will happen next and explicitly announcing task completion, such as, ‘I’ll see you tomorrow… That’s all done now’. These strategies help prevent confusion [50].

#### Barriers

One significant barrier is the use of ‘elderspeak’, common in hospital and care home settings. This includes using childish phrases (‘Are your feetsies [feet] hot still?’), diminutives (‘pumpkin’), collective pronouns (‘Oh, that’s not something we wanna [want to] do’) and belittling language (‘Let’s put your leash on’), which is often counterproductive and associated with increased resistance to care [46, 48]. Despite this, some practitioners believe that using pet names is appreciated by PWD [42].

Overloading PWD with excessive information or instructions can be overwhelming and hinder communication [67]. Asking specific recall questions can also cause distress [55, 57, 58]. Using jargon, acronyms or complex language can create barriers, given the language comprehension difficulties in dementia [53]. This can be particularly problematic in end-of-life discussions, where clear and sensitive communication is crucial [56]. Language barriers, including differences in dialect, can also inhibit communication [56, 66]. In hospital outpatient settings, patients were less satisfied when doctors made pronouncements about treatment options (‘I will start you on medication’) without offering choices [54]. Finally, ambiguous conversational closings such as ‘See you soon’, or open-ended questions like ‘Is there anything else?’ can cause confusion and uncertainty about how to respond, resulting in communication breakdowns [50].

### Nonverbal communication techniques

Nonverbal communication is vital in interacting with PWD. Physical contact, like a pat on the shoulder or a hug, offers reassurance and establishes rapport for effective communication [42, 58, 67]. Paying attention to prosodic features—such as tone, intonation and speech rate—ensures communication is attuned to people’s emotional and cognitive needs [60]. Showing empathy by mirroring the person’s body language further deepens the connection [55]. Gestures like pointing or demonstrating actions help direct a person’s focus to specific tasks or objects, while tactile prompts, such as handing an object or guided touch, capture attention and aid understanding [62].

Creating an atmosphere conducive to communication is also achieved through eye contact and appropriate facial expressions [52, 64]. Practitioners should maintain a relaxed, happy and confident attitude, as this positively impacts how PWD feel about their care [60].

Another technique is centring, where practitioners focus on the present moment and remain fully attentive to PWD. This, combined with active listening, showing genuine interest and closely observing the person’s behaviour, allows practitioners to gather information and respond effectively [65]. Allowing PWD to set the rhythm of the dialogue by giving ample time to respond [35, 38, 64, 65] and following their lead when they initiate a conversation helps maintain respectful interaction [67].

Being attuned to the nonverbal cues used by PWD is crucial, as these signals are often highly individualised and require careful interpretation [57]. Conversely, inappropriate interactions from professionals, such as using a commanding tone, being inattentive or ignoring the person’s signals, can lead to PWD feeling discomfort, pain or sadness, which may go unaddressed [67].

### Use of external aids

Visual memory aids, memory books and patient passports with pictures and words can help PWD recall and express important information about themselves and engage in meaningful interactions [37, 38, 40, 42]. Images can also support PWD in understanding questions about their social and leisure-based preferences [39]. Guidance tools, such as the Conversation Starter Kit and the Serious Illness Conversation Guide (SICG-D) provide structured frameworks for ACP conversations, enhance engagement of PWD and guide practitioners in keeping discussions focused and responding effectively to emotions [36, 59]. The physical environment can also stimulate interactions; for example, turning on a light in the morning can encourage participation in daily activities [62]. Themed corridors in care homes can act as conversation starters [58], or personal objects at home can serve as cues to discussions [60].

## Support for the workforce

Practitioners often find communication with PWD challenging due to expressive and receptive language problems, memory issues and lack of interactional reciprocity. Difficulty in understanding people’s communication attempts can lead to fear and a lack of confidence in interactions [60, 65]. These psychological effects highlight the importance of providing opportunities for practitioners to discuss challenges and receive support for their wellbeing [60].

Staff need comprehensive training on communication in dementia [38], covering strategies for interacting with PWD who have limited verbal language skills, as well as coping strategies to prevent burnout [63, 66]. Education on dementia progression, including end of life, enables more effective and sensitive communication [56]. Finally, it is important to ensure that staff have the confidence to manage specialist questions on dementia [51].

## Discussion

### Key findings

Our narrative synthesis presented three key themes in communication strategies in dementia care: understanding the person and their care context, the use of communication techniques and support for the workforce. These findings uncover practical strategies, such as linguistic elements, tone, body language and emotional, relational aspects like understanding the individual.

The themes revealed an interesting comparison between the studies’ foci. Qualitative studies focusing on the views of PWD and carers emphasise the importance of knowing the person and forming meaningful relationships as critical communication components of personalised care [41, 51, 53, 56]. This aligns closely with Kitwood’s concept of personhood [8] and Brooker’s VIPS framework [9]. In contrast, practitioner-focused studies highlight practical communication strategies to maintain efficiency and clarity during care tasks [58, 62]. Some studies use the term ‘resistance’, which can imply that PWD are to blame for difficulties in task completion [49, 50, 62]. However, other studies emphasise how verbal and nonverbal techniques alongside care task completion can contribute to a person-centred approach [44, 61]. Balancing meaningful conversation with care task–related talk has been highlighted in a previous review [20] as a trainable skill, where care home staff–resident communication improved following training to embed communication strategies in daily care activities.

The perceived effectiveness of communication strategies varies; for example, the use of pet names is viewed differently by practitioners and PWD, with some seeing it as endearing and others as infantilising [42, 46]. Many included findings reflect the practitioner perspectives rather than those of PWD or their carers, potentially leading to a skewed understanding.

Overlap between identified themes suggests effective communication in dementia care relies on multiple strategies, combining language-based strategies and relational, person-centred methods [13, 68] as well as a mix of verbal and nonverbal techniques [62, 67]. Within each theme, language-based strategies and relational aspects interact. In ‘Understanding the person and care context’, combining task-focused communication with nontask-related talk is highlighted as beneficial for relationship building. In Communication Strategies, using language-based strategies helps foster respect and deepen personal connections, and ‘Support for the workforce’ suggests that a lack of confidence and competence in using communication strategies can lead to fear and avoidance of meaningful interaction, potentially increasing isolation for people with dementia. This aligns with findings from a review of website content on communication strategies in dementia [22] with themes identified incorporating both transactional and relationship-building communication elements. In line with our findings on workforce support, only 61% of communication strategies shared on websites were evidence-based, suggesting that formal and informal caregivers need support selecting and implementing strategies that are most likely to be effective [22].

Most studies, when severity is specified, focus on individuals with moderate to severe dementia, except for two focusing on mild dementia [47, 54]. This distinction is important, as communication strategies may vary depending on severity. Individuals with moderate dementia might benefit from verbal techniques, whereas those with severe dementia may require greater emphasis on nonverbal behaviour [62].

The review shows a predominance of studies conducted in care homes, where communication strategies are tailored to managing daily care tasks [57]. These task-oriented strategies may not translate well to other contexts where the emphasis might be on care planning and coordination. In care homes, staff may have time to get to know residents, unlike primary care, where time impacts the relational aspect of communication, although capacity and staffing issues are relevant in both settings.

A significant evidence gap is the limited exploration of cultural and language barriers, with few studies addressing this [56, 66]. As dementia care increasingly involves individuals from diverse cultural backgrounds, this gap is concerning.

### Strengths and limitations

This review provides a comprehensive overview, including a wide range of study designs and care settings for a nuanced understanding of the complexities involved in dementia care communication. However, most research is from high-income countries and focuses on care home settings, limiting generalisability to other care settings and to low- and middle-income countries.

The quality of the included studies varied. Relational themes, such as understanding the person and family, were primarily drawn from qualitative studies, which generally had strong methodologies, despite often lacking discussion on researcher reflexivity. Some of these studies also described practical communication strategies.

‘Practical themes, including verbal and nonverbal communication’ and the use of external aids, were mostly identified in quantitative studies assessing specific interventions. However, these studies often had methodological limitations, such as a lack of control groups, inadequate randomisation procedures and failure to address confounding factors. Despite these limitations, practical themes remain valuable, as this review focuses on identifying available strategies rather than evaluating their effectiveness*.*

### Implications and recommendations

The review highlights a substantial gap in the support provided to the workforce. There is a clear need for comprehensive training programmes that address both the technical aspects of communication and the psychological challenges of fear and lack of confidence in interaction faced by HCPs. Future research should prioritise the development and evaluation of communication strategies that are adaptable to various care settings. There is a need for more research in primary care settings, especially given the current policy emphasis on increasing the role of primary care in postdiagnostic dementia support [12]. More research is needed to explore communication strategies in people at all stages of the dementia trajectory and in diverse cultural contexts, acknowledging differences within and across ethnicities and cultural backgrounds. Finally, more research that incorporates the perspectives of PWD and their carers is necessary to ensure that communication strategies align with their needs and preferences.

## Conclusion

This systematic review has identified key communication strategies for delivering personalised dementia care, including understanding the person and their care context, employing both verbal and nonverbal communication techniques and using external aids and ensuring support for the workforce. It highlights the importance of combining both practical and emotional approaches. Future research and policy should focus on enhancing and expanding these communication strategies, particularly in areas such as primary care and culturally diverse contexts, while improving support for the workforce. Such efforts will help ensure better outcomes for PWD and their carers.

## Supplementary Material

SUPPLEMENTARY_DATA_afaf120
