# Supplementary material for: Communication strategies for delivering personalised dementia care and support: a mixed-methods systematic review and narrative synthesis
Source: Age Ageing. 2025 May 15;54(5):afaf120. doi: 10.1093/ageing/afaf120 (PMC12078768; doi:10.1093/ageing/afaf120)
Supplement: SUPPLEMENTARY_DATA_afaf120 [file supplementary_data_afaf120.docx]

## **CONTENTS**

**Appendix 1. Search Strategies**

**Appendix 2. Full Data Extraction Table**

**Appendix 3. Quality Assessment of Included Studies**

Table C.1 Quality Assessment of Qualitative Studies

Table C.2 Quality Assessment of Quasi-Experimental Studies

Table C.3 Quality Assessment of Cross-Sectional Studies

Table C.4 Quality Assessment of Randomised Controlled Trials

Table C.5 Quality Assessment of Mixed Method Studies

## **Appendix 1. Search Strategies**

| **Database** | **Subject Headings** |  | **Keywords** |
| --- | --- | --- | --- |
| **MEDLINE** | Dementia | OR | dementia*  alzheimer* |
|  | AND | | |
|  | Advance Care Planning  Patient Care Planning  Case Management  Patient-Centered Care | OR | patient* cent?red care  person* cent?red care  personali*ed care  advance* care plan*  patient* care plan* |
|  | AND | | |
|  | Communication  Health Communication  Professional-patient relations  Physician-patient relations  Nurse-patient relations | OR | communicat*  discuss*  talk*  discours*  interact*  convers*  dialog*  chat* |
| **EMBASE** | Dementia | OR | dementia*  alzheimer* |
|  | AND | | |
|  | Advance Care Planning  Patient Care Planning  Patient Decision Making  Case Management | OR | patient* cent?red care  person* cent?red care  personali*ed care  advance* care plan*  patient* care plan* |
|  | AND | | |
|  | Interpersonal communication  Professional-patient relationship  Doctor-patient relationship  Nurse-patient relationship | OR | communicat*  discuss*  talk*  discours*  interact*  convers*  dialog*  chat* |
| **EMcare** | Dementia | OR | dementia*  alzheimer* |
|  | AND | | |
|  | Advance Care Planning  Patient Care Planning  Patient Decision Making  Case Management | OR | patient* cent?red care  person* cent?red care  personali*ed care  advance* care plan*  patient* care plan* |
|  | AND | | |
|  | Interpersonal communication  Professional-patient relationship  Doctor-patient relationship  Nurse-patient relationship | OR | communicat*  discuss*  talk*  discours*  interact*  convers*  dialog*  chat* |
| **PsycINFO** | Dementia | OR | dementia*  alzheimer* |
|  | AND | | |
|  | Treatment Planning  Patient centered care  Case Management | OR | patient* cent?red care  person* cent?red care  personali*ed care  advance* care plan*  patient* care plan* |
|  | AND | | |
|  | Interpersonal communication | OR | communicat*  discuss*  talk*  discours*  interact*  convers*  dialog*  chat* |
| **CINAHL** | Dementia | OR | dementia*  alzheimer* |
|  | AND | | |
|  | Patient Centered Care  Patient Care Plans  Advance Care Planning  Case Management  Decision Making, Patient | OR | patient* cent*red care  person* cent*red care  personali*ed care  advance* care plan*  patient* care plan* |
|  | AND | | |
|  | Communication  Professional-Patient Relations  Physician-Patient Relations  Nurse-Patient Relations | OR | communicat*  discuss*  talk*  discours*  interact*  convers*  dialog*  chat* |
| **Web of Science** | N/A |  | dementia*  alzheimer* |
|  | AND | | |
|  | N/A |  | patient* cent?red care  person* cent?red care  personali*ed care  advance* care plan*  patient* care plan* |
|  | AND | | |
|  | N/A |  | communicat*  discuss*  talk*  discours*  interact*  convers*  dialog*  chat* |
| **Scopus** | N/A |  | dementia*  alzheimer* |
|  | AND | | |
|  | N/A |  | “patient* cent*red care”  “person* cent*red care  “personali*ed care”  “advance* care plan*”  “patient* care plan*” |
|  | AND | | |
|  | N/A |  | communicat*  discuss*  talk*  discours*  interact*  convers*  dialog*  chat* |

## **Appendix 2. Full Data Extraction Table**

| **Author (Year)** | **Title** | **Country, Care Setting** | **Aim** | **Study Design** | **Patient/Carer Demographics** | **Dementia Severity** | **Practitioner Demographics** | **Sample Size** | **Communication Strategies** | **Main Findings** |
| --- | --- | --- | --- | --- | --- | --- | --- | --- | --- | --- |
| **Acton et al. (2007)** | **Increasing social communication in persons with dementia** | USA, Care home | To test the effect of individualised communication prescriptions on social communication between persons with dementia (PWD) and nurse. | Quasi-experimental (pre-test/post-test, no control group) | Age: 76-88 (mean 81) Gender: female (9), male (1) | MMSE score Severe: 2 participants Moderate: 6 participants Mild: 2 participants | N/A | 10 residents | Individualised communication prescription developed based on analysis of the first interview with each PWD and implemented during the second interview. Development included communication abilities, demographic information, and likes and dislikes. Example strategies: 1. Avoid open leads ('Is there anything that you would like to talk with me about today?') 2. Use focused leads: a conversational cue that opens conversations and suggests a specific direction or subject ('We certainly have had interesting weather lately, haven't we.') 3. Allow extra time for response 4. Use minimal cues: used by the listener that he/she is engaged in the conversation but does not contribute to the subject of conversation ('yes', 'OK', head nod) 5. Avoid yes/no, one-word answer, and specific recall questions | Communication prescriptions had a positive effect on social communication between PWD and nurse. 1. Total number of subjects’ words did not increase from the first to the second interview 2. Average of subjects’ words per topic (527 words for interview 1 and 788 words for interview 2; t = –2.332, df = 9, p < .05) 3. The percentage of topics introduced by the subjects increased 4. Total number of topics needed to sustain the interview decreased from 204 in interview 1 to 145 in interview 2 (t = 2.385, df = 9, p < .05) 6. Participants with the lowest MMSE scores showed the most improvement |
| **Allwood et al. (2017)** | **Should I stay or should I go? How HCPs close encounters with people with dementia in the acute hospital setting** | UK, Hospital inpatient | 1. To examine how HCPs close encounters with people with dementia in the acute hospital setting. 2. To examine barriers to achieving closing in this setting and contrast this with primary care. | Qualitative (conversation analysis) | 26 patients  Specific demographics not provided | Not reported | 9 doctors, 11 nurses (including mental health nurses), 6 allied health professionals or AHPs (physiotherapists, speech and language therapists, and occupational therapists) | 26 patients, 9 doctors, 11 nurses, 6 AHPs (41 video-recorded encounters) | 1. Open-ended pre-closings ( 'can I do anything for you?' ‘Anything you want to ask me before I go?’ 'do you want a hand with anything else while I'm here?') 2. Mixed messages (healthcare practitioners appears to work towards closing an encounter, but then opens up the interaction again) 3. Non-specific and indeterminate terms ('soon' 'I'll be around') | 1. Open-ended questions can lead to interactional difficulty in acute setting, in contrast to primary care setting. PWDs appear to have problem with understanding the purpose and scope of such questions in the context of an encounter that they have not initiated, and they may not fully comprehend being acutely unwell and in hospital. 2. Mixed-message cause confusion for patient, they may continue to orient to the task as completed, and produce strong resistance. 3. Concrete arrangement is preferrable compared to non-specific language and indeterminate terms. |
| **Alnes et al. (2011)** | **Marte Meo Counselling: a promising tool to support positive interactions between residents with dementia and nurses in nursing homes** | Norway, Care home | To investigate whether changes could be identified in the interactions between people with dementia and their nurses during morning care following Marte Meo Counselling (MMC). | Quasi-experimental (with matched control group) | Gender: All female Age: 71-87 | Moderate to severe | Professions: 7 enrolled nurses (ENs), 6 registered nurses (RNs) Gender: All female Age: 36-61 years (mean 51) Experience in dementia care: 3-30 years (mean 9.3) | 10 residents,13 nurses | Function Supporting Elements (FSEs) 1. Prepare for a good beginning and a positive atmosphere through tone and eye contact 2. Locate, confirm and follow the person’s focus 3. State what is happening, what is going to happen, and what is experienced 4. Reinforce coping ability by providing help to start and end an activity 5. Help the resident to be in rhythm in the dialogue by waiting for an answer or supporting the resident’s initiatives 6. Help or support the resident to respond to new people or situations in the setting 7. Pay attention to physical contact 8. Lead in a positive way  Inappropriate interaction (II) 1. Information is inaccurate, unnecessary or excessive considering situation and resident’s level of cognitive functioning 2. Memory challenge given to resident, e.g. asked about things that obviously cannot remember 3. Nurse carrying out ADL that resident is capable of doing 4. Nurse advises resident in a commanding tone 5. Nurse gives the resident multiple instructions or carries out several activities at the same time 6. Nurse is inattentive or ignores signals from resident 7. Pace not adjusted to resident’s capability 8. Potentially degrading comment from nurse 9. Irrelevant talk (nurse speaks about something that is not relevant in the context) 10. Resident expresses pain, discomfort, anger, sadness without receiving a response from the nurse | 1. The cases in the intervention group were stable or scored higher on FSEs after the intervention, compared to the control group. 2. In the intervention group, II is either low or somewhat reduced in the videos following the intervention. 3. The control group shows greater variation within the same case in terms of both FSE and II. 4. II was more pronounced in the control group. 5. MMC helped nurses become more aware of residents' capabilities and how to support them effectively, leading to increased self-confidence in their caregiving role |
| **Anantapong et al. (2022)** | **Communication between the multidisciplinary team and families regarding nutrition and hydration for people with severe dementia in acute hospitals: a qualitative study** | UK, Hospital inpatient | To understand the experiences, views and needs of family carers and hospital staff about communication and discussions of nutrition and hydration for people with severe dementia during hospital admission. | Qualitative (semi-structured interviews) | Family carers Age: 29-78 (mean 53.2) Gender: Female (10), Male (2) Ethnicity: Asian (2), Black (1), White British (7), White Other (2) Relationship: Daughter/son (10), spouse (1), friend (1) | Severe | Hospital staff Age: 28-54 (mean 38.9) Gender: Female (16), Male (1) Ethnicity: Asian (4), Black (0), White British (11), White Irish (1), White Other (1) Professions: Dietician (1), Psychologist (1), Nurse (5), Physician (3), Speech and Language Therapist (7) | 29 (12 family carers and 17 staff) | Potential strategies to the conversations about nutrition and hydration in acute hospitals: 1. Observe or speak to the person with dementia first 2. Identify an appropriate family member and hospital staff 3. Have early and ongoing discussions 4. Involve multidisciplinary team including palliative care team 5. Discuss overall dementia progression and goals of care 6. Review current treatments 7. Build dialogues on information from and understanding of the person with dementia and family carers 8. Encourage validation and use empathic reassurance 9. Have honest discussions but be aware of varying levels of readiness 10. Be sensitive to emotions and values 11. Set clear steps ahead (consider treatment escalation plans) 12. Ensure clear documentation and information sharing 13. Explain and reassure continuing care plan for the end of life | Themes developed from interviews: 1. Prerequisites to initiating communication about eating and drinking (staff needed to gain confidence and knowledge before starting, family carers saw this as a delay or poor communication) 2. Communication aiming to develop agreed care plans (multidisciplinary team approach seen as positive among staff but seen as lack of consistency from family carers) 3. Difficulty discussing palliative and end-of-life care (many families did not understand that dementia was a life-limiting disease and the roles of palliative care team) 4. Needs of information and plans about future eating and drinking difficulties (many family carers did not receive enough information before hospital discharge |
| **Baillie et al. (2012)** | **Caring for older people with dementia in hospital. Part two: strategies** | UK, Hospital inpatient | To explore adult nursing students’ experiences of caring for older people with dementia in acute hospital settings. | Qualitative (focus groups) | N/A | Not reported | Adult nursing students who had had at least one practice placement  Specific demographics not provided | 4 focus groups, 4-6 students in each group | 1. Getting to know the person and building a relationship 2. Involvement of families 3. Flexible and creative care approaches (e.g allowing a patient to sit at the nurses’ station for social interaction or adapting mealtime routines to accommodate patients' preferences) 4. Comfort and communication: - the need to be calm and keep tone of voice and facial expression kind - understanding the person - provide familiarity - clear information and explanations - use reassurance proactively | 1. The strategies described seemed congruent with a person-centred approach: get to know them, promoting continuation of self and normality, enabling familiar activities.  2. Students often had to negotiate these approaches to fit in with hospital routines. |
| **Barbosa et al. (2016)** | **Effects of a psycho-educational intervention on direct care workers' communicative behaviors with residents with dementia** | Portugal, Care home | To assess the effects of a person-centred care-based psycho-educational intervention on Direct Care Workers (DCWs) verbal and nonverbal communicative behaviours with residents with dementia during morning care. | Quasi-experimental (pre-test post-test control group design) | 47 residents participated Specific demographics not described | Moderate to severe dementia (DSM IV) | Direct Care Workers (DCWs) Gender: All female Age: mean 44.72 years (SD 9.02) Marital status: Married (67.2%), Widowed (5.2%), Single (6.9%), Divorced/Separated (15.5%), Other (5.2%) Education: Primary school (25.9%), Middle school (20.7%), High school (41.4%), College degree (1.7%), Other (10.3%) | 58 DCWs: 27 in the experiment group and 31 in the control group Residents: 47 | The intervention included two components: educative and supportive. 1. Educative component: information about PCC and dementia, communicative behavioural strategies to interact with residents with dementia (eg, give simple choices, use validation, allows time to respond, use individual’s name and eye contact), Information about challenging behaviours and strategies to deal with them, strategies to enhance the physical and social environment for the person with dementia (eg, decrease background noise; post signs as reminders), motor stimulation strategies (e.g. encourage the person to perform one task or a part of it), and multisensory stimulation strategies (e.g., provide a gentle massage while washing resident’s hair). 2. Supportive component: provided DCWs with coping strategies to manage work-related stress and prevent burnout (e.g., time management and problem solving), relaxation techniques (e.g., abdominal breathing and guided imagery), stretching and strengthening exercises were practiced. | 1. The PE intervention had a broader positive impact on DCWs' communicative behaviours compared to the education-only intervention. 2. Within the experimental group there was a positive change from pre- to post-test on the frequency of all DCWs’ communicative behaviours. |
| **Barbosa et al. (2016)** | **Effects of a Psychoeducational Intervention for Direct Care Workers Caring for People With Dementia: Results From a 6-Month Follow-Up Study** | Portugal, Care home | To examine the 6-month effects of a person-centred care (PCC)-based psycho-educational (PE) intervention on Direct Care Workers (DCWs) verbal and nonverbal communicative behaviours with residents with dementia during morning care. | Quasi-experimental (pre-test post-test control group design) | 47 residents participated Specific demographics not described | Moderate to severe dementia (DSM IV) | Direct Care Workers (DCWs) Gender: All female Age: mean 44.72 years (SD 9.02) Marital status: Married (67.2%), Widowed (5.2%), Single (6.9%), Divorced/Separated (15.5%), Other (5.2%) Education: Primary school (25.9%), Middle school (20.7%), High school (41.4%), College degree (1.7%), Other (10.3%) | 58 DCWs: 27 in the experiment group and 31 in the control group Residents: 47 | The intervention included two components: educative and supportive. 1. Educative component: information about PCC and dementia, communicative behavioural strategies to interact with residents with dementia (eg, give simple choices, use validation, allows time to respond, use individual’s name and eye contact), Information about challenging behaviours and strategies to deal with them, strategies to enhance the physical and social environment for the person with dementia (eg, decrease background noise; post signs as reminders), motor stimulation strategies (e.g. encourage the person to perform one task or a part of it), and multisensory stimulation strategies (e.g., provide a gentle massage while washing resident’s hair). 2. Supportive component: provided DCWs with coping strategies to manage work-related stress and prevent burnout (e.g., time management and problem solving), relaxation techniques (e.g., abdominal breathing and guided imagery), stretching and strengthening exercises were practiced. | Within the intervention group, the frequency of the majority (24 of 26 behaviours) of person-centred behaviours improved immediately after the intervention (T2). However, 22 of 26 behaviours dropped at 6-month follow-up. For 10 of these behaviours, the frequencies were lower than those found at baseline. |
| **Benbow et al. (2011)** | **Patients' and carers' views on dementia workforce skills** | UK, Community | To identify the skills which people living with a dementia and carers felt needed to be developed in the health and social care workforce | Qualitative (feedback form and interview) | 11 people living with dementia 11 carers’ group members 47 from dementia cafe (29 from one cafe, 18 from a second) were cafe attenders, who were mostly (but not exclusively) carers | Not reported | N/A | 69 participants | List of core competencies related to communication: 1. Knowledge about dementia 2. Person centred care 3. Communication 4. Relationships 5. Support for carers 6. Helping people engage in activities | 1. Knowledge about dementia needs to be communicated to people living with dementia to avoid marginalising them. Need to balance theoretical knowledge with real-life experiences (emotional awareness). 2. Many staff did not tailor care offered to the individual and family receiving care. Consistency in caregiving staff was highlighted as crucial for building trust and reducing distress. 3. Communication includes having the opportunity to talk with someone about their fears, hopes and challenges. Clear, jargon-free communication was emphasised. 4. People living with a dementia highlighted the need for a more positive personal relationship with those caring for them |
| **Berry et al. (2023)** | **Adapting the Serious Illness Conversation Guide for Dementia Care** | USA, Mixed | To adapt the Serious Illness Conversation Guide (SICG) for use in dementia care to promote high-quality advance care planning (ACP) conversations. | Mixed method (survey and interview) | Patients Age: 65-89 years (mean 77.43) Gender: male (7), female (7)  Caregivers Age: 27-75 years (mean 65.4) Gender: male (10), female (8) Relationships: Spouse/partner (12), sibling (2), adult child (3), friend (1) | Not reported | N/A | 32 participants (14 patients and 18 caregivers) | Alteration to the original SICG: 1. Phrasing to reflect patient-caregiver dyad 2. Dementia-specific language 3. Establishment of caregiver/medical proxy dialogue 4. Dementia specific end-of-life care 5. Caregiver-directed questions 6. Caregiver-directed support phrases  SICG-D outline: Setup "I'd like to talk about what is ahead with your/your__'s illness and do some thinking in advance about what is important to you/your ___ so that I can make sure we provide the best care possible - is this okay?" Assess "What is your understanding now of your/your__'s illness?" Share "I want to share with you my understanding of where things are with you/your__'s illness..." Explore "What are you/your___'s most important goals if their health worsens?" Close "I've heard you say that___ is important to you/your___. Keeping that in mind, and what we know about you/your___'s illness, I recommend ___. How does this plan seem to you?" Interstitial skills: the Wish/Worry/Wonder framework, responding to emotion (Name, Understand, Respect, Support, Explore), conversation connectors (achieve thematic saturation, paraphrase/summarise, normalisation of extremes, parking lot/bookmarking) | 1. 94% of survey respondents reported a positive impression of the conversation facilitated by the SICG-D and 89% endorsed the incorporation of the adapted guide into dementia healthcare. 2. The adapted guide was found to promote values-based ACP conversations and facilitated effective triadic communication between patients, caregivers, and clinicians. |
| **Bourgeois et al. (2001)** | **Memory aids as an augmentative and alternative communication strategy for nursing home residents with dementia** | USA, Care home | To examine the effect of memory aids on conversations between nursing aides and residents with dementia. | Cluster Randomised Controlled Trial (RCT) | Treatment Group Age: mean 85.73 years (SD=5.21) Gender: female (88%), male (12%) Race: Caucasian (85%), African American (15%) Education: mean 12.91 years (SD=5.21)  Control Group Age: mean 84.27 years (SD=6.61) Gender: female (76%), male (24%) Race: Caucasian (76%), African American (21%), Hispanic (3%) Education: mean 13.16 years (SD=3.39) | MMSE Score:  Treatment group: mean 11.85 (SD=6.9) Control group: mean 13.06 (SD=7.13) | Treatment Group Age: Mean 35.39 years (SD=6.97) Gender: female (94%), male (6%) Ethnicity: Caucasian (3%), African American (97%) Education: mean 13.7 years (SD=1.16)  Control Group Age: Mean 37.3 years (SD=6.71) Gender: female (79%), male (21%) Ethnicity: Caucasian (3%), African American (3%), Asian (6%) Education: mean 14.3 years (SD=0.53) | 66 residents and 66 nursing aides | Contents of memory books: 1. Autobiographical information: Each memory book contained personal information about the resident, including their name, family members, significant life events, hobbies, and interests. 2. Daily schedules: Information on the daily routines for which the resident needed some assistance, such as bathing. 3. Problem resolution: The answer to repetitive question such as 'When is lunch?' 4. Each sentence/page was illustrated with photographs, drawings, or graphics. The pages were laminated, hole punched, held together with 3/4-inch rings, and worn by the resident on a necklace or belt.  How memory books are used: 1. As memory cues during care activities. Example: For bathing, aides would open the book to the bathing page, show it to the resident, and announce bath time. 2. As distraction tools during care when residents were uncooperative. Example: Aides could open the book to a page about the resident’s family, direct their attention to the picture, and ask a question about the family while continuing the care activity. 3. Books could be used at other times and in other settings to facilitate cooperation, encourage conversation and distract the resident. | 1. Quantity of verbal interactions: - Residents in the treatment group were observed using their memory books during conversations 83.2% of the time (SD = 27.4). - Residents in the treatment group used more utterances per conversation post-treatment compared to the control group. - Nursing aides in the treatment group had increased the number of utterances in post-training conversations compared to control staff. 2. Quality of verbal interactions: - Residents in the treatment group made significantly more informative utterances when they used their memory aids, whereas both groups made fewer uninformative utterances over time. 3. Impact on quality of life: - Residents in both groups consistently rated themselves as less depressed than nursing aides rated them at both times. - There were no significant effects of the intervention on depression ratings over time for either residents or nursing aides. - After training, nursing aides in treatment group rated residents as less depressed than at baseline, and their ratings more closely approximated the residents’ ratings, in contrast to the control group. |
| **Bourgeois et al. (2004)** | **Communication skills training for nursing aides of residents with dementia** | USA, Care home | To investigate the effectiveness of specific components of a communication skills intervention program and the amount of training effort required to teach and maintain each skill for 3-4 months post-training. | Quasi-experimental (two-group comparison study) | Treatment Group Age: mean 84.17 years (SD=6.57) Gender: female (81%), male (19%) Race: White (85.7%), Black (14.3%) Education: mean 12.75 years (SD=3.39)  Control Group Age: mean 84.75 years (SD=7.19) Gender: female (79%), male (21%) Race: White (72.6%), Black (25.8%), Hispanic (1.6%) Education: mean 11.91 years (SD=3.86) | MMSE Score:  Treatment group: mean 11.79 (SD=6.52) Control group: mean 11.73 (SD=6.73) | Nursing Aides (NA) 1. Treatment Group Age: Mean 35.15 years (SD=7.70) Gender: female (93%), male (7%) Ethnicity: White (8.8%), Black (91.2%) Education: mean 13.96 years (SD=0.91) 2. Control Group Age: Mean 33.76 years (SD=8.19) Gender: female (88.4%), male (11.6%) Ethnicity: White (5.8%), Black (92.8%), Asian (1.4%) Education: mean 14.12 years (SD=0.71)  LPN (Treatment group only)  Age: Mean 37.67 years (SD=9.49)  Gender: female (91.3%), male (8.7%)  Ethnicity: White (39.1%), Black (56.5%), Asian (4.3%)  Education: mean 14.83 years (SD=0.92) | 126 nursing aides (57 treatment and 69 control group), 125 residents (63 treatment group; 62 control group), 23 LPNs (treatment group only) | Training programme components: 1. a didactic in-service to present content about effective communication and memory aids, behaviours related to dementia and strategies for responding to these behaviors effectively 2. one-on-one, criterion-based training to apply the new strategies with residents during care interactions 3. the use of memory books with residents as a memory aid and instructional tool during care interactions 4. a staff management system including self-monitoring and supervisory feedback  Effective skills: - Announce care when entering the resident's room - Address the resident by name - Introduce self by name - Give appropriate announcement for every activity - Wait 5 seconds before providing physical help Effective instructions: - Give short and clear instructions - Give positive feedback when the resident follows directions - Talk about the resident’s life or day - Use memory books to explain care Ineffective instructions: - Multi-step instructions - Negative statements - Unhelpful questions | 1. Knowledge assessment: No significant changes on knowledge test score pre and post inservice (p > .20). 2. NA care interaction skills: - NAs showed low rates of effective skills at baseline except for "Announce Care" and "Address Resident by Name" (used 70-80% of the time). - Significant difference from baseline to training and post-training measurement for effective skills (announce care, address the resident by name, introduce self by name, announce every ADL, wait 5 s before helping), effective instructions (use short and clear instructions, use positive feedback, social talk about resident's life). No significant differences were found for unhelpful statements or multi-step instructions. - At the 3-month follow-up, seven of the eight trained behaviours maintained or exceeded post-training rates, with only Introduce Self declining but not to baseline levels. 3. Memory Books: - Memory books were present 84.3% of the time during training phase and 26.5% during the follow-up phase. - Memory books were used an average of 1.45 seconds during 5-minute care interactions. 4. Generalised training effects on communicative interactions:  - Positive statements increased from 1.69 at baseline to 3.53 post-training for the treatment group. - NA verbal interaction duration increased from 25.61 seconds at baseline to 37.53 seconds post-training for the treatment group. - Positive correlations at post-training between positive feedback and NA verbal interaction (Pearson’s r = .397, p < .05) and number of short instructions and NA verbal interaction. 5. Trained NAs maintained skill use 3 months post-training, though some skills showed a decline from post-training levels but remained above baseline. |
| **Burshnic and Bourgeois (2022)** | **A Seat at the Table: Supporting Persons with Severe Dementia in Communicating Their Preferences** | USA, Care home | To examine the impact of two assessment conditions (standard verbal and externally supported) on the consistency of preferences over time and utterance types in response to preference questions. | Quasi-experimental (within-subjects design) | Gender: all female Age: mean 89 (SD = 7.55) Ethnicity: Caucasian (18), African American (1) Residential: assisted living (memory care) communities (11), nursing homes (10) | Average BIMS score of 4 (SD = 2.00). | N/A | 21 residents | Standard Verbal Condition: 1. Residents responded verbally to preference questions (34 questions on social contact, personal development, and leisure activities) 2. Three preference rating options: Very Important, Somewhat Important, Not Important.  Visual-and-Text Supported Condition: 1. Preference questions paired with photographs and simple text 2. Responses facilitated through a sorting mat labelled with the three preference ratings. | 1. Results of paired-samples t-tests showed no significant difference between percentage agreement (consistency) for standard and supported conditions (p = .181) 2. Residents made significantly fewer requests for clarification in the supported condition compared to the standard condition (p = .007). This finding suggests comprehension of preference questions improved with use of supported assessment methods. 3. No significant differences in acknowledgments, elaborations, or off-topic utterances between the conditions |
| **Dooley et al. (2018)** | **Involving patients with dementia in decisions to initiate treatment: Effect on patient acceptance, satisfaction and medication prescription** | UK, Hospital outpatient | 1. To examine how doctors involve patients with dementia in decisions to start medication and how this affects patient acceptance of medication. 2. To assess any association between doctors recommendation with patient cognitive function, satisfaction and prescription. | Quantitative (cross-sectional) | Patient: Gender: Female (44), Male (27) Age: 65-91 (mean 81) Ethnicity: White British/Irish (59), White other (3), Caribbean (3), Black (1), African (1), Other (2), Missing (2) Diagnosis: Alzheimer's disease (69%), Mixed dementia (20%), Dementia unspecified (5%), Parkinson’s disease (3%), Lewy body dementia (3%) Capacity to make medication decisions: Full (61%), Partial (22%), None (4%), Missing (13%)  Companion: Spouse/partner (27) Child/child in law (27) Sibling (2) Friend (2) Other (8) Missing (1) | Addenbrooke’s Cognitive Examination – III (mean score: 69, range: 41-94) MMSE (mean score: 23, range: 15-28) | Professional type: Consultant psychiatrist (15), consultant geriatrician (3), specialty doctor (3) Gender: Female (11), Male (10) Ethnicity: White British (14), White other (3), Asian or Asian British (2), Indian (2) Years of experience in dementia: 1-25 years (mean 12 years) | 71 dementia patients with 67 companions and 21 doctors across 71 medication recommendation discussions. | Treatment recommendation: 1. Pronouncements, where patients are given no choice (‘I will start you on medication’) 2. Proposals, where patients are invited to endorse or collaborate with the doctor’s idea (‘How about trying medication?’) 3. Suggestions, where medication is endorsed by doctors but patients are given the choice (‘Would you like to try medication?’) 4. Offers, where doctors show willingness to prescribe for the patient but do not actively endorse medication (‘Do you want me to give you medication?’) 5. Assertions, where doctors state the fact that medication exists without endorsement or explicit recommendation (‘There is a medication’) | 1. Suggestions were used in 42% of meetings, proposals 25%, pronouncements 11%, offers 9%, and assertions 13%. 2. 63% of patients passively resisted medication, 18.5% actively resisted, and 18.5% explicitly accepted. 3. There was a significant association between recommendation format and patient response (Fisher’s exact test P = 0.014). Active resistance occurred only after suggestions or proposals; pronouncements always led to passive resistance. 4. Subtle differences in the recommendation have an effect on patient response. With proposals, doctors are inviting patients to join in with their endorsement of medication. With suggestions, doctors are inviting the patient to decide whether they would like to start medication. Proposals lead to higher levels of acceptance whereas suggestions lead to higher levels of resistance. 4. No association between recommendation format and cognitive impairment level (ACE-III: p = 0.751, MMSE: p = 0.660). 5. Patients were significantly less satisfied with pronouncements (mean satisfaction score: 14.3/20) compared to other formats (16.5-17.3/20) (p = 0.024). 6. No association between patient acceptance/resistance and prescription outcome (p = 0.561). Medication was just as often prescribed when patients resisted as when they accepted. If medication was not prescribed, this was usually because of a need for further investigation or doctors planning to put other support systems in place before starting medication. |
| **Douglas and MacPherson (2021)** | **Positive Changes in Certified Nursing Assistants’ Communication Behaviors With People With Dementia: Feasibility of a Coaching Strategy** | USA, Care home | 1. Investigate whether a 6-week coaching strategy resulted in positive changes in self-perceived knowledge and efficacy and positive communication behaviours in certified nursing assistants (CNAs) working with people with dementia in a skilled nursing facility. 2. Assess the impact of the coaching strategy on negative responsive behaviours of people with dementia. | Quasi-experimental (Pre-test/post-test observational, single-subject, multiple-baseline study) | Age: 74 to 102 Varying degrees of communication and reading abilities (font size for reading ranged from 12 to 72 points) | Not reported | Certified Nursing Assistants (CNAs) with experience ranging from 6 months to 25 years in skilled nursing facilities, minimal training in dementia communication strategies. | 7 CNAs, 7 people with dementia | Coaching strategy focused on positive communication behaviours: 1. Approaching the person with dementia from the front, gaining eye contact, and smiling 2. Greeting the individual using their name 3. Introducing oneself and the activity 4. Showing an external memory support for the activity 5. Gently guiding the person with dementia to an activity | 1. Six out of seven CNAs improved their in self-perceived knowledge and efficacy from pre- to post-coaching, but the difference was not statistically significant. 2. Statistically significant increase in positive communication behaviours from baseline to follow-up. 3. Statistically significant decrease in overall responsive behaviours of people with dementia from pre- to post-coaching. |
| **Kaasalainen et al. (2021)** | **Evaluating the Implementation of the Conversation Starter Kit in Long Term Care** | Canada, Care home | To evaluate whether the use of Conversation Starter Kit (CSK) booklet improve long-term care home residents' engagement in advance care planning (ACP) | Quasi-experimental (one group pre/post design) | Residents Age: Average 77.3 years (SD: 11.87) Gender: Female (29), Male (26) Marital Status: Widowed (23), Married/Common-law (12), Never married (10), Divorced/Separated (9), Prefer not to answer (1) Ethnicity: White (35), Chinese (3), Black (1), Filipino (1), North American Indian (3), Jewish (3), Other (10) CHESS Score: Score 0 (30), indicating low to no health instability  Family members Average age: 62.82 years (SD: 14.91) Gender: Female (24), Male (13) Relationship to Resident: Children (24), Spouse (7), Sibling (7) Ethnicity: White (34), Other (3) Education: Bachelor's degree (17), Post-grad/Professional degree (10), High school/Trade/Apprenticeship/No high school (10) | Not reported | N/A | 55 residents, 11 family members paired with decision-making residents (DMRs), 24 family members paired with non-decision-making residents (non-DMRs) | Conversation Starter Kit (CSK) booklet: Step 1: Get Ready Step 2: Get Set Step 3: Go Step 4: Keep Going The first two steps (Get Ready & Get Set) help promote reflection and learning about how to engage with a family member in ACP discussion through tips, prompts, and both closed- and open-ended questions that focus on values, wishes, concerns, and preferences for future care. The last two components (Go & Keep Going) focus on moving forward to future discussions with family/friends and health care providers.  Complete booklet available from: https://theconversationproject.org/wp-content/uploads/2016/09/TCP_NEWStarterKit_Writable_Sept2016_FINAL.pdf | 1. Residents reported higher engagement scores after completing the booklet than before on all 4 subscales of ACP Engagement Survey (Decision Maker, Quality of Life, Flexibility, Ask Questions). 2. The family members reported average self-efficacy score decreased to after having completed the CSK booklet. Probably due to learning about the type of decisions that would need to be made in the future and realised that CSK is just the beginning. 3. The importance of residents and families completing the CSK booklet together to promote discussion. CSK booklet should be introduced early, and staff need to support families and residents to have this discussion together. 3. Residents and family members mostly agreed the information and guidance in the booklet was presented clearly and was easy to understand. However, some stated that the language and terms used were too "wordy", confusing, and impersonal. 5. Those who did not complete the booklet felt that it was not useful, did not agree with or enjoy the content, circumstances no longer enabled them to participate in, or too difficult to understand. |
| **Kamalraj et al. (2021)** | **Communication in home care: Understanding the lived experiences of formal caregivers communicating with persons living with dementia** | Canada, Care in community | To understand the lived experiences of formal caregivers, specifically Personal Support Workers (PSWs), communicating with people living with dementia who live in their own homes. | Qualitative (hermeneutic phenomenological, semi-structured interview) | N/A | Not reported | Personal Support Workers (PSWs) Gender: female (13) male (2) Age: mean 35.3 years (22-58) Ethnicity: White (12), Black/African-Canadian (2), Asian (1) Education: High school (1), College (14) Experience in Home Care: mean 6.8 years (0.7–20.2) Hours/Week in Home Care: mean 38.1 hours (22–70) Number of Clients: 1-5 (4), 6-10 (4), >10 (7) | 15 PSWs | 1. Positive communication strategies such as using an appropriate rate of speech. 2. Negative communication strategies such as directive communication and threatening PWD with repercussions for their actions. 3. Non-verbal communication (body language and attitude) such as exhibiting a ‘relaxed’, ‘happy’, and confident attitude, and being attentive to one's own physical behaviour, prosodic features of speech production. 4. Home environment can be used to facilitate successful communication. | 1. Challenges: - Dementia-related impairments as barriers (impaired verbal language production, problems with topic management, verbal repetition, slurred speech, reversion to a native language, memory loss, confusion, repetitive actions, reverting to the past, wandering, etc) - Emotional toll of communication (they had to repeat themselves, fear of clients who exhibited verbally aggressive behaviour, unpredictability of dementia, lack of confidence) - Consequences of communication breakdowns (anger or verbal aggression, lack of reciprocity) 2. Valuing communication in care - Treating communication as a need, a crucial aspect of care. Clients enjoyed participating in conversation, regardless of their impairments. Talking while providing care offered clients a sense of security. - Being self-aware and self-reflective about positive and negative communication strategies and the reaction from PWD, and the importance of non-verbal communication. 3. Home is a personal space - The dual nature of families' presence and involvement. Positive role of families such as interpreting and persuading PWDs, and providing information to PSWs. Family involvement could complicate the communication experience by making awkward situation and limit the effort of PSW to better understand PWD. - Availability of environmental cues such as personal objects can facilitate successful communication. |
| **Mundadan et al. (2023)** | **Language-Based Strategies that Support Person-Centered Communication in Formal Home Care Interactions with Persons Living with Dementia** | Canada, Care in community | To investigate the overlap between language-based strategies and person-centred communication (PCC) strategies and missed opportunities for PCC in home care setting with persons living with dementia. | Cross-sectional | Gender: Female (7) Male (5) Ethnicity: all White Age: 87.8 (77-97) | Vascular dementia secondary to stroke: 1 Mild cognitive impairment: 1 Suspected Alzheimer's: 2 Diagnosed with dementia and had probable Alzheimer's: 8 | Personal Support Workers (PSWs) Gender: all female Ethnicity: White (10) Black/African Canadian (1) Age: 47.2 (21-62) | 12 residents, 11 PSWs | Positive person work in Person-centred Communication (PCC): 1. Recognition: includes acknowledging the person living with dementia as an individual, including calling them by name and integrating their life story into conversation ('Is that Lucy's husband?'). 2. Negotiation: includes communication that consults on needs, desires, and preferences.('What would you like for breakfast?') 3. Validation: includes communication that is feelings oriented and affirms the person living with dementia. ('Well that's why we help each other') 4. Facilitation: includes communication used to initiate and sustain interactions, such as positive instruction to facilitate client's completion of a task. ('You run some water to wash your upper body') 5. Missed opportunities for PCC: occur where one of the above indicators could have been used to support the client’s personhood, but were absent 6. Missed opportunity alternative: occurs when a caregiver uses a non-person-centred alternative during an opportunity to be person-centred 7. Missed opportunity omission is when a nonresponse or minimal response, such as failing to greet the client or allowing enough time to respond, occurred in place of a person-centred response.  Effective language-based strategies: 1. Verbatim and paraphrased repetitions, using nouns instead of pronouns, right-branching sentences, positive instructions, and allowing time to respond to facilitate comprehensions. 2. Yes/no questions, unfinished sentence prompts, minimal turns, matching comments, and affirmations can improve expression. 3. Announce activity/intent clearly, ask for permission, and use politeness and affirmations to prompt collaboration 4. Verification questions and comments, giving more information, and filling in missing information can help resolve issues related to speech production, language structure/processing, cognition, or hearing. 5. Addressing the person living with dementia by name/title and using greetings | 1. Several language-based strategies support PCC goals: - Yes/No Questions: Overlapped with three of the four PCC indicators (recognition, negotiation, facilitation) at a frequency greater than 10%. - Affirmations and Positive Feedback: Demonstrate validation. - Addressing by Name/Title: Shows recognition. - Announcements of Action/Intent: Facilitate actions when used appropriately. 2. Many missed opportunities overlapped with language-based strategies. - Announcements of Action/Intent: Often used without prior consultation, reducing individual preference consideration. - Positive Instructions: Can become patronizing if not mindful of the client's autonomy. - Yes/No Questions: Should be balanced with other question types to avoid limiting the client's responses. 3. Home care settings allow for more person-centred language due to more one-on-one interaction time compared to long-term care. Home care interactions often involve personal conversation, leading to higher PCC. 4. Caregivers should be mindful communicators, using pauses and varied question types to support both comprehension and meaningful participation. |
| **Palmer (2012)** | **Caregivers' Desired Patterns of Communication with Nursing Home Staff** | USA, Care home | To explore the communication patterns of caregivers who place a family member with Alzheimer’s disease (AD) in a nursing home (NH) and to identify desired patterns of communication with NH staff. | Qualitative (hermeneutic phenomenological, interview) | Family caregivers Age: Adult daughters: Mean 56.7 years (SD: 1.27) Spouses: Mean 70.8 years (SD: 7.55) Gender: Adult daughters (6) Spouses (4 husbands, 5 wives) Ethnicity: Predominantly White, with only one participant from a minority group | Not reported | N/A | 15 family caregivers | Six desired patterns of communication (TALKKK): 1. Tell: Caregivers desire to be informed about the resident's daily activities, health status, and any changes in condition. 2. Ask: NH staff should ask caregivers for their expert advice regarding the needs, habits, routines, and behaviours of the resident. 3. Listen: Caregivers want NH staff to listen to their concerns and knowledge about the resident and respond to their input. 4. Know: Caregivers expect NH staff to get to know the residents personally, including their individual needs and preferences. 5. Be Knowledgeable: NH staff should be knowledgeable about Alzheimer's disease, its progression, and dementia-specific care. 6. Share Knowledge: NH staff should share relevant knowledge with caregivers, including information about medications and care strategies. | 1. The desired patterns of communication (TALKKK) in the previous column were generated through qualitative analysis 2. By knowing and implementing the preferred patterns of communication, nurses and NH staff convey essential information to caregivers and optimize time spent with them. 3. Acknowledging caregivers as partners in care, respecting them as experts in knowing the resident, and communicating with them accordingly promotes trust that safe and effective nursing care will be provided in their absence. |
| **Passalacqua and Harwood (2012)** | **VIPS Communication Skills Training for Paraprofessional Dementia Caregivers: An Intervention to Increase Person-Centered Dementia Care** | USA, Care home | To examine the feasibility of a communication intervention for caregivers based explicitly on Brooker’s (2004) four elements (VIPS) of person-centred dementia care and to see whether the intervention fostered more person-centred attitudes, beliefs, and communication behaviours. | Quasi-experimental (pre-test/post-test design) | N/A | Not reported | Facility caregivers Gender: female (823), male (3) Age: 18 to 30 (12), 31 to 49 (7) 50 or older (7) Ethnicity data were not gathered on the sample, but of the caregivers at the facility, approximately 35% South or East Asian, 35% Hispanic, 15% White, and 15% Black. | 26 facility caregivers | VIPS Model: 1. Valuing People (V): Respectful communication, avoiding elderspeak, recognising the rights and value of residents. 2. Individualized Care (I): Tailoring care based on residents’ unique personalities, histories, and preferences. 3. Personal Perspectives (P): Understanding and empathising with the resident’s perspective and experiences. Nonverbal techniques (e.g., face residents at their level, utilize touch) and verbal techniques (e.g., use concrete language, repeat keywords) for better communication. Indirect repair occurs when a listener repeats or rephrases an apparently “incorrect” statement by someone with dementia, with the primary goal of maintaining conversational flow rather than “correcting.”  4. Social Environment (S): providing a positive social environment for interaction. The use of memory aids (e.g., memory books) and alternatives and supplements to verbal communication (e.g., nonverbal communication, writing, and illustration) were explored. | 1. Following the workshops, caregivers reported less depersonalisation of residents (p<0.05), more hope for Alzheimer’s patients (p<0.01), and more empathy (p<0.10). 2. Caregivers reported using more gestures (p<0.05), more humour (p<0.10), asking more yes/no questions (p<0.05), and giving the choice between two options (p<0.05) more after the workshops as compared to before. 3. The attempt to persuade caregivers to reduce addressing residents by "pet names" raised notable resistance among the staff, they insisted that residents enjoy pet names. |
| **Riachi (2017)** | **Person-centred communication in dementia care: a qualitative study of the use of the SPECAL method by care workers in the UK** | UK, Care in community | To investigate the use of the SPECAL™ (Specialized Early Care for Alzheimer’s) communication techniques by care workers in maintaining well-being in clients with dementia. | Qualitative (semi-structured interview) | N/A | Not reported | Gender: all female Work roles: domiciliary care worker (5) senior manager and trainer (2) Dementia care work experience: 8 years (2), 4 years (3), 10+ years (1), 2 years (1) | 7 staff | The SPECAL™ (Specialized Early Care for Alzheimer’s) communication techniques composed of: 1. Three Golden Rules: - Avoid posing questions to the person with dementia - Listen to the person with dementia and learn from them - Do not contradict the person with dementia 2. Primary Theme: those who know the client best are consulted to uncover what things or experiences made the client happiest in their pre-dementia past to develop a vocabulary to signal to the client (for example, it’s time for dinner). 3. Recognising client personhood: care should be individually tailored. 4. Techniques: - Protecting: care workers used communication techniques to avoid distressing the client, such as avoiding direct questions, steering clear of topics that could cause anxiety, using indirect suggestions and body language to guide clients without causing stress. - Reassuring: verbal and non-verbal techniques to communicate a sense of safety, comfort and familiarity. Not contradicting, giving praise to the client, using repeated positive message provided constant reassurance for the clients. Using "mind games" to help clients perform tasks without stress. - Empathising: care workers built close relationships with clients through validation, active listening, and role-playing, expressing empathy when client was exhibiting signs of withdrawal or anxiety. - Reflecting: Closely observing and adapting to the client's changing symptoms and responses. | 1. The primary goal identified was "Maintaining client personhood," reflecting care workers' efforts to uphold the individuality and avoiding interactions that could undermine self-esteem. 2. Care workers adapted their communication techniques described in the previous column. 3. Regular supervision and support from managers trained in SPECAL™ were crucial for the effective use of these techniques. |
| **Saini et al. (2016)** | **An ethnographic study of strategies to support discussions with family members on end-of-life care for people with advanced dementia in nursing homes** | UK, Care home | 1. To examine practices relating to end-of-life discussions with family members of people with advanced dementia residing in nursing homes. 2. To explore strategies for improving practice in end-of-life care discussions. | Ethnographic study (reflective diary and semi-structured interview) | Family members Relationship: Daughters (2) Husband (1) Son (1) Age: 54-76 Ethnicity: all white British | Not reported | Independent Care Liaison (ICL) HCPs: managers (2), activity co-ordinators (2), deputy managers (3) nurses (2), healthcare assistants (6), GP (2), palliative care nurse (1), geriatrician (1) | Diary: 1 ICL Interview: 19 HCPs 4 Family members | 1. Educating family and staff about dementia progression and EOL care - Families and staff needing and wanting more information about diagnosis, symptoms and progression of dementia - NH staff lacking confidence to initiate and have EOL conversations - Staff attributing symptoms and behaviours to dementia without trying to identify an underlying cause - Training and case scenarios increasing staff confidence and being able to see things from the families’ perspective - Discussions with family appear to increase their capacity to make informed decisions, eg around cardiopulmonary resuscitation - Family sessions generated much discussion and appeared a good avenue for education - Usefulness of written information to support discussions - Importance of ICL as a role model to staff in having conversations with family and communicating with residents with advanced dementia 2. Appreciating the value of in-depth EOL discussions (over documentation) - Importance of ongoing dialogue with family to build relationships, provide reassurance and allow time for family to process information - NH staff prioritising documentation such as DNAR or not for hospitalisation over ongoing dialogue – task oriented approach and not appreciating the complexity and need for individualised approach to these discussions - Importance of addressing family member’s current issues and concerns before discussing future plans - Need to acknowledge family members’ grief and guilt - Difficulties communicating in English prohibit in-depth and sensitive conversations about EOL 3. Providing time and space for sensitive discussions - Not suitable having sensitive conversations with family in communal areas such as lounge or dining room - Spending sufficient time with family to address their questions and explore their concerns – including follow-up sessions/ongoing dialogue. The ICL was able to provide this time. - NH staff and GP having multiple demands preventing spending focused and uninterrupted time with family 4. Having an independent HCP or team with responsibility for EOL discussions - ICL role was independent from GP and NH and considered to be primarily in interests of resident and family - Independent person provides alternative and fresh view of the residents’ needs and care | 1. Current practices explained in the previous column. 2. Consistent findings between diary content and interview: - Families often lack understanding of dementia progression, which hampers EOL care discussions - Staff need more education and confidence to discuss EOL care effectively 3. Inconsistent findings: - Cultural issues within nursing homes that interviews did not capture, such as staff being task-driven rather than fostering ongoing dialogue with families |
| **Savundranayagam et al. (2014)** | **Missed opportunities for person-centered communication: Implications for staff-resident interactions in long-term care** | USA, Care home | 1. To assess whether staff–resident interactions during routine caregiving tasks were person-centred. 2. To assess the extent to which staff miss opportunities to use person-centred communication strategies. | Cross sectional (measurement of utterances in conversation) | Gender: all female except 2 | MMSE score / AD stage Middle stage (12-18): 35% Late stage (0-11): 65% | Gender: all female except 1 | 13 staff-resident dyads | Person-centred Communication based on Kitwood (1997): 1. Recognition: incorporating their life histories into conversation 2. Negotiation: consulting a resident about his/her preferences, desires, and needs. Particularly useful with persons in the later stage of dementia, where they may not be able to communicate verbally their needs or their understanding. 3. Facilitation: recognizing when the person with dementia attempts an action or a thought and enabling them to complete actions they would otherwise be unable to do. 4. Validation: expressing and understanding the feelings of the resident with dementia.  Missed opportunities: Instances where person-centred communication could have been used but was not. | 1. The average proportion of staff utterances that were person-centred in each conversation transcript was 0.36. There were no differences in the proportion with residents with middle or late stage AD. The majority of the interaction were task focused, suggesting that person-centred communication is possible during care tasks. 2. The average proportion of missed opportunities for person-centred communication was 0.11. There were no differences between middle or late stage AD. 3. The missed opportunities were categorised as categorized as (a) person-centred alternatives to original staff utterances (40%), and (b) omissions, where a person-centred utterance could have been used during a conversation but was not present (60%). 4. Context for missed opportunities: a. Recognition: commonly occurred when staff began and ended their interaction (eg. staff did not address the resident by their name when greeting and leaving). b. Negotiation: staff told residents what to do without providing options or not inviting their help in completing the task. c. Facilitation: - when resident was confused about their environment - when staff failed to probe or find out more about resident when s/he mentioned something of value to him/her - when resident had a passive role (when the task is not cumbersome for the staff, such as combing hair, asking the resident about her/his day or the activities s/he is interested in illustrates staff interest in the resident’s life d. Validation: - when staff failed to affirm resident's feelings - when residents expressed lack of confidence or self-deprecating comments - when residents expressed positive emotions 5. It is possible that once staff are trained to identify person-centred strategies they use currently, they may be more likely to sustain person-centred interactions. |
| **Savundranayagam and Moore-Nielsen (2015)** | **Language-based communication strategies that support person-centered communication with persons with dementia** | USA, Care home | To examine whether language-based strategies for effective communication with persons with dementia overlap with indicators of person-centred communication | Cross-sectional (measurement of utterances in conversation) | Gender: all female except 2 | MMSE score / AD stage Middle stage (12-18): 35% Late stage (0-11): 65% | Gender: all female except 1 | 13 staff-resident dyads | Person-centred Communication: 1. Recognition 2. Negotiation 3. Facilitation 4. Validation  Language based strategies: 1. Completion of turns (timing): give ample time for person to respond; do not interrupt; the social partner gives the speaker time to complete their thoughts. 2. Announces intent clearly: inform a person about a topic change. 3. Confirms understanding: through restatements of what the resident said: summary of prior talk that communicates what is worth highlighting. 4. Confirm understanding by asking for clarification: this includes giving choices of what you think he/she means 5. Statements that inform the person exactly what is misunderstood 6. Rephrase to add clarity to a previous statement 7. Verbatim repetition 8. Ask the other person to repeat what he/she said 9. Open-ended questions that rely on semantic memory only; do not rely on episodic memory 10. Choice questions that rely on semantic memory only; and that do not rely on episodic memory 11. Yes/No questions that rely on semantic memory only; do not rely on episodic memory 12. Unfinished sentences that the interactant is encouraged to complete 13. Matching comment 14. Matching association: statements that offer one’s own opinion or some information about personal experiences 15. Newsmarks: emphasize the noteworthiness of the prior turn for the recipient 16. Affirmations: Statements that display agreements or acknowledge feelings and are often used with requests or instructions 17. Politeness to address resistiveness 18. Greeting 19. Right-branching sentences 20. Place modifiers after nouns 21. Place modifiers after verbs | 1. There were overlaps between language-based strategies and person-centred communication. Recognition: 26% greetings, 21% affirmations, 13% questions (yes/no, open-ended), 15% rephrasing Negotiation: 74% questions (yes/no, choice, open-ended) Facilitation: 51% questions (yes/no, open-ended, choice), 21% affirmations, 13% rephrasing Validation: 89% affirmations 2. Staff need training to use more diverse language strategies to support the personhood of residents with dementia. 3. Similar language strategies are employed across the spectrum of interactions ranging from task-based to conversational. |
| **Savundranayagam et al. (2016)** | **Resident Reactions to Person-Centered Communication by Long-Term Care Staff** | USA, Care home | To examine resident reactions in response to conversations involving person-centred communication and missed opportunities for such communication by staff during routine care tasks. | Cross-sectional | Gender: all female except 2 | MMSE score / AD stage Middle stage (12-18): 35% Late stage (0-11): 65% | Gender: all female except 1 | 13 staff-resident dyads | Person-centred Communication: 1. Recognition: incorporating their life histories into conversation 2. Negotiation: consulting a resident about his/her preferences, desires, and needs. Particularly useful with persons in the later stage of dementia, where they may not be able to communicate verbally their needs or their understanding. 3. Facilitation: recognizing when the person with dementia attempts an action or a thought and enabling them to complete actions they would otherwise be unable to do. 4. Validation: expressing and understanding the feelings of the resident with dementia.  Missed opportunities: Instances where person-centred communication could have been used but was not.  Resident reactions: 1. Positive: cooperation, self-disclosure, going along with conversation, asking for clarification, and politeness 2. Negative: resistiveness to care and distress | 1. Increased use of person-centred communication by staff resulted in more positive resident reactions (P < 0.001) 2. Missed opportunities for person-centred communication led to negative resident reactions (P < 0.05) 3. Examples of Resident Reactions: - Cooperation: Residents complied and agreed with staff instructions when negotiation was used. - Self-Disclosure: Residents shared biographical information when staff facilitated conversation. - Going Along With Conversation: Residents contributed more when staff welcomed and facilitated communication. - Asking for Clarification: Residents initiated communication repair to understand staff better. - Politeness: Residents expressed gratitude, praise, and encouragement towards staff. - Resistiveness to Care: Negative reactions to directive language and lack of consideration of resident preferences and comfort. - Distress: Expressions of pain, discomfort, or anxiety when staff did not address their needs. |
| **Shaw et al. (2022)** | **Characteristics of elderspeak communication in hospital dementia care: Findings from The Nurse Talk observational study** | USA, Hospital inpatient | 1. To describe attributes of elderspeak use in hospital dementia care. 2. To determine what characteristics are associated with nursing staff use of elderspeak communication with hospitalised patients with dementia. | Cross-sectional | Gender: male (9), female (7) Ethnicity: non-Hispanic White (15), other (1) Background: community-dwellers (7), coming from long-term care (9) Diagnosis: Alzheimer’s disease (5), unspecified dementia (6), and other dementias (5) | Moderately severe dementia (12) | Role: staff nurses (27) and nursing assistants (26) Gender: female (45), male (8) Ethnicity: White (43), other (10) Experience: Less than five years in healthcare (33), relatively new to their hospital unit (mean = 2.2 years, SD = 3.3) Education: Higher education levels among staff nurses compared to nursing assistants. Staff nurses scored higher on knowledge of dementia (KIDE scale). | 53 staff nurses and nursing assistants 16 patients with dementia 88 care encounters audio-recorded | The elderspeak category includes three non-mutually exclusive major categories (i.e., semantics, discourse, and prosody) and 11 non-mutually exclusive subcategories. 1. The semantics (infantilizing word choices) - childish terms ('I know, you’re kind of a roly poly today, aren’t ya?') - diminutives ('pumpkin', 'sweetie', 'dear') - collective pronouns ('Okay, do we wanna stay on the bed pan a little longer?') - short words or phrases subcategories. 2. The discourse category (types of speech that reflect an imbalance between the communicative goals of conveying care, respect, and control) - directive/imperative phrases - exaggerated praise - tag questions - minimizing words or mitigating expressions ('It’ll be really quick, just a little pinch, it’ll be okay') - reflective phrases - interruptions - laughing at or belittling ('Are you gettin cold, Frank? [chuckles]') 3. The prosody category (altered prosodic, or intonational, patterns such as a raised pitch, a sing-song pattern of intonation, excessive changes in pitch range or volume, or over-articulation) | 1. Elderspeak was identified in 85 of the 88 all care encounters (96.6%). On average, elderspeak comprised 11.7% SD=10.4% of the observation time and 28.7% SD=21.0% of the nursing staff communication. 2. Particularly common attributes: minimizing words and mitigating expressions (87.5%), diminutives (29.6%), childish terms/phrases (44.3%), collective pronoun substitution (42%), prosodic alterations (73.9%). 3. Older staff nurses used elderspeak more frequently than younger staff. Elderspeak use increased by 12.5% with patients experiencing delirium and by 1.5% for each additional day the patient was hospitalized. 4. Non-significant factors are dementia severity, comorbidity severity, gender, race/ethnicity of nursing staff, confidence in dementia care, and knowledge of dementia. |
| **Sánchez-Martínez et al. (2023)** | **"Now I Understand You": Changes in the Communication of Professionals in Nursing Homes After Receiving Training in the Validation Method** | Spain, Care home | 1. To explore communication barriers between professionals and people with dementia in nursing homes. 2. To observe changes in communication patterns after professionals received training in the Validation Method (BVM). | Quasi-experimental (mixed methods) | N/A | Not reported | Gender: female (11) Age: mean 32 (SD=12) Education: professional training (4), occupational training (4), higher education (3) Role: Nurse's aide (8), physiotherapist (1), Social educator (2) | 11 professionals, 33 interviews (each participant interviewed 3 times) | The validation method is a specific method of communication for older people with dementia. It is based on three essential factors: (a) professional attitude based on respect and empathy (b) verbal and nonverbal communication techniques (c) theoretical framework and principles based on humanism, psychoanalytic theory, and developmental theory to better understand the reasons behind the behaviour of older people with dementia  Communication strategies: 1. Non-verbal techniques: - Centring: Professionals focused on the present moment and fully attended to the person with dementia. - Empathy: Showing genuine interest and understanding of the emotions of people with dementia. - Observing: Paying close attention to the behaviour and reactions of people with dementia to gather more information. 2. Verbal techniques: - Ambiguity: Using nonspecific pronouns to navigate conversations when the meaning is unclear (e.g., he, she, that). - Trusted Listening: Sincerely listening to patients, helping them express their needs and emotions (e.g., connecting with life stories). - Reminiscence: Recalling memories to help patients manage situations (e.g., discussing past experiences with music). - Rephrasing: Repeating key aspects of patients' communication to understand them better. | 1. Pretraining phase (T0) : a. Communication barriers related to PWDs: challenging behaviours, difficulties in the language of the person with dementia, memory problems b. Communication barriers related to the professionals: lack of tools or training, difficulties in understanding the meaning of residents' behaviour, organisational difficulties (not having enough time to have good communication), ignorance of the resident's life history c. Emotional reactions to communication difficulties: frustration, stress, sadness, impotence, distrust in their professionalism 2. Post training analysis (T1-T2) a. Changes in communication strategies (3 non-verbal and 4 verbal) described in the previous column b. Changes in attitudes - Broader vision: Professionals better understood the meaning behind patients' behaviours, moving away from stereotypical judgments - Humanisation: Focus shifted from the dementia diagnosis to the individual person c. Emotional reactions - More reflective about their roles, reducing feelings of impotence and distrust in their skills - Increased satisfaction and pride in their work - Training introduced new challenges, causing some stress |
| **Song et al. (2019)** | **Can Persons with Dementia Meaningfully Participate in Advance Care Planning Discussions? A Mixed-Methods Study of SPIRIT** | USA, Hospital outpatient | 1. To adapt SPIRIT (sharing patient's illness representations to increase trust) intervention for PWDs and their surrogates  2. To assess whether PWDs could meaningfully participate in the SPIRIT intervention and complete outcome assessment | Mixed methods study (quantitative with qualitative interviews) | PWDs Age: Mean age 74.2 years (SD = 7.6) Gender: male (6), female (17) Ethnicity: non-Hispanic White (17), other (6) Dementia Type: Alzheimer’s disease (18), Lewy bodies (2), vascular dementia (1), frontotemporal dementia (1), mixed dementia (1)  Surrogates Age: Mean age 63.1 years (SD = 11.8) for in-person, 69.7 years (SD = 9.8) for remote Gender: female (15), male (8) Ethnicity: White (non-Hispanic) (18), other (5) Relationship to PWD: Spouse/partner (17), child (6) | Mild dementia (9) Moderate dementia (14, 60%) | N/A | 23 dyads (PWDs and their surrogates) | Steps of the SPIRIT Intervention: 1. Assessing illness representation: Understanding the cognitive, emotional, and spiritual aspects of the patient’s ideas about their illness. 2. Identifying gaps and concerns: Identifying areas where the patient and surrogate have misunderstandings or concerns. 3. Creating conditions for conceptual change: Facilitating conditions that encourage the patient to rethink their illness representation. 4. Introducing replacement information: Providing new information to replace misconceptions or knowledge gaps. 5. Summarizing: Summarizing the discussion to ensure understanding and retention. 6. Setting goals and planning: Completing a Goals-of-Care tool to indicate patient’s preferences and planning accordingly.  Adaptations for PWDs: 1. Enhanced consent techniques to the delivery process 2. Segmented information delivery 3. Verification of comprehension | 1. All 23 PWDs were able to articulate their values and end-of-life wishes somewhat or very coherently. The adapted SPIRIT intervention appears to have enabled PWDs to engage in an ACP discussion and to promote the authenticity of exchanges about experiences surrounding illness and values. 2. Decision-making capacity may be the more critical mental faculty, rather than global cognitive function in ACP discussions. 3. Longer duration compared with earlier SPIRIT studies because the interventionist was required to speak slowly, repeat questions for the PWD, and ask clarifying questions. |
| **Wang et al. (2013)** | **Long-term Care Nurses' Communication Difficulties with People Living with Dementia in Taiwan** | Taiwan, Care home | To explore the communication difficulties faced by nurses when interacting with patients who have dementia. | Qualitative (Phenomenological approach) | N/A | Middle to late-stage dementia | Gender: All female Age: 24 to 35 years old. Education: Bachelor of Science in Nursing (7), Associate Degree in Nursing (6), Vocational Nursing Diploma (2) Experience: 6 months to 7 years Nursing Levels: N1 (9), N2 (3), N3 (3). Work Settings: 8 in residential care facilities, 4 in assisted living facilities, 3 in nursing homes | 15 nurses | 1. Different language: difference in dialect between the nurse and the patient. - Repetitive responses: occurs when patients and nurses repeat their messages due to memory and cognitive impairments. Example: a nurse describes repeatedly answering a patient's question about the time of day, resulting in the patient becoming angry. - Lack of language consensus: occurs when there is no common language or understanding between nurses and patients. Example: patients refusing to eat despite nurses' repeated efforts to persuade them. 2. Blocked messages: the inability of nurses to understand the true underlying meaning of the information - Difficulty in accessing emotions: nurses struggle to interpret the emotional states of patients who have lost verbal language skills. Example: a nurse describes difficulty in making a patient smile or open up emotionally. - Difficulty in understanding needs: nurses find it challenging to comprehend and respond to the behavioural messages of patients with impaired verbal expression. Example: a nurse describes confusion when a patient claims to have a stomachache but then denies pain shortly afterward. | 1. Two different themes emerged from this study: different language and blocked messages. 2. Nurses are more task-oriented. When nurses focus their communication efforts toward self-perspective and routine care, and patients fail to cooperate, ineffective communication occurs. |
| **Ward et al. (2008)** | **A different story: Exploring patterns of communication in residential dementia care** | UK, Mixed | 1. To explore communication patterns in dementia-care settings and their impact on care quality. 2. To understand the role of communication in the care of residents with dementia and to evaluate current care practices. | Qualitative (video, diary, semi-structured interview) | Gender: male (2), female (15) | Not reported | 32 care staff and 6 non-care staff Job Roles: care assistants, night care workers, group leaders, qualified nurses, home managers, assistant managers, and non-care workers (domestic staff, administrators, maintenance workers, laundry assistants, and dining-room coordinators) | 38 staff 17 residents | Care-speak is a distinct style and pattern of speech used by care workers when performing a task. This style of speech was characterised by a series of directives, framed by narration of the task at hand and punctuated by words of encouragement. Four main components of care-speak: 1. an opening 2. signal of intent 3. task accomplishment 4. closing remark or gesture When a resident failed to comply with a task, the staff responded with: 1. desistance (i.e. desisting from or postponing a task) 2. verbal persuasion 3. coalition with a second or even a third carer 4. enforcement using authoritative commands or physical presence | 1. Only 2.5% of resident's day involves contact with care workers.  2. Interactions were 77% task-based, 15% social or relationship-oriented, and 8% combining both. Most encounters were initiated by staff. 3. Only approximately one-third of the contact time between care staff and residents involved verbal exchanges. 4. Care-speak is featured in task-based encounters. This limits input by the resident, there was little space during the contact for talk about non-task topics. 5. Care workers maintain order in dementia-care settings, restricting residents' movement and discouraging interaction. This orderliness creates artificial barriers to communication, which may not be in line with residents' wishes. 6. Care workers lacked support to develop communication skills with people with dementia, leading to social exclusion. |
| **Wheeler and Oyebode (2010)** | **Dementia care 1: Person centred approaches help to promote effective communication** | UK, Care home | To gather care home staff views on communication issues and explore strategies to improve communication skills. | Qualitative (Focus group discussions) | N/A | Not reported | Not described | 36 direct care staff members | Staff to Resident communication: 1. Talking while doing: Engaging residents during routine tasks; using everyday activities as opportunities for interaction. 2. Reminiscence: Using past memories to engage residents and enhance personhood. Social history can provide possible clues to explain why residents displayed certain behaviours and alter staff perceptions of residents' competency. 3. Empowerment: Involving residents in care planning; recognizing their capacity to voice opinions and desires. 4. Disempowerment: Task-focused care led to residents displaying challenging behaviours. 5. Reassurance and love: Providing constant reassurance to residents, give them comfort, stability, and security.  Staff to Family Communication: 1. Involving families: Homes operated open-door policies, encouraging families to view the home as an extension of their own. Families provided valuable social history information, aiding in care planning. 2. Keeping family informed: Regular communication about residents' health status and changes in care plans. 3. Conflicts sometimes arose from differing opinions on care. | 1. Staff to Resident Communication: - Positive interactions based on person-centred approaches improved resident engagement and reduced challenging behaviours. - Time constraints often limited opportunities for meaningful interactions. - Task-oriented approaches led to disempowerment and increased challenging behaviours. 2. Staff to Family Communication: - Family involvement in care planning was inconsistent across homes. - Effective communication with families was linked to higher satisfaction and better care outcomes. 3. Homes adopting person-centred approaches empowered residents and improved care quality. |
| **Williams et al. (2009)** | **Elderspeak communication: impact on dementia care** | USA, Care home | To examine the relationship between elderspeak communication by nursing staff and resistiveness to care behaviours in residents with dementia during activities of daily living (ADL). | Cross-sectional | Age: mean 82.9 years (69-97) Gender: female (15), male (5) Ethnicity: Caucasian (19), African American (1) ADL Functional Score: Mean 29.6 (range 7-52) | MDS-COGS scores ranged from 4 to 9 (mean 6.4), indicating moderate dementia | Age: Mean 35 years (21-54) Gender: female (43), male (9) Ethnicity: White (35), African American (15), Pacific Islander (1), Hispanic or Latino (2) Roles: Primarily certified nursing assistants (CNAs) (78%), with nurses, therapists, and social workers also included Experience: Mean 7.5 years in patient care (range 0.25-31 years), mean 3.5 years in current facility (range 0.10-18 years) | 52 nursing staff and 20 residents with dementia, 80 interactions during ADL care | Elderspeak communication: Simplistic vocabulary and grammar, shortened sentences, slowed speech, elevated pitch and volume, inappropriate terms of endearment or diminutive (e.g., 'honey', 'good girl'), collective pronoun substitutions (e.g., 'Are we ready for our bath?'), tag questions (e.g., 'You want to get up now, don’t you?') | 1. Significant increase in the probability of RTC when elderspeak was used compared with normal talk and silence. 2. Elderspeak use did not vary significantly across different ADL activities except for collective pronoun use, which was higher during bathing and dressing. 3. Nursing staff were more likely to use elderspeak during neutral resident behaviour than during resistiveness to care or cooperative behaviour. |
| **Williams et al. (2017)** | **A Communication Intervention to Reduce Resistiveness in Dementia Care: A Cluster Randomized Controlled Trial** | USA, Care home | To evaluate whether the Changing Talk (CHAT) intervention, aimed at improving nursing home (NH) staff communication by reducing elderspeak, would reduce resistiveness to care (RTC) behaviours in residents with dementia. | Cluster RCT | Age: Mean 87.7 years (72-104) Gender: female (21), male (6) Race: White (27) Ethnicity: Non-Hispanic (26), Hispanic (1) Comorbidity Score: Mean 27.2 (SD = 5.3) Depression: None (18), Mild (4), Moderate (2), Moderately severe (1) RTC: Behaviour not exhibited (19), Occurred 1-3 days (5), Occurred 4-6 days (2) | Dementia stage: Moderate impairment (17), Severe impairment (10)  Communication ability: Understands (5), Usually understands (13), Sometimes understands (5), Rarely/never understands (3) | Age: Mean 36.7 years (21-67) Gender: female (26), male (3) Race: White (25), African American (3), Native American (1) Ethnicity: Non-Hispanic (25) Education: High school (14), College (15) Experience as caregiver: Mean 10.9 years (SD = 10.2) Experience in current facility: Mean 4.2 years (SD = 5.9) | 42 dyads (29 staff and 27 residents) | CHAT Intervention: 1. Three 1-hour sessions provided to staff to reduce elderspeak and improve effective communication. 2. Sessions focused on self-monitoring and avoiding elderspeak, understanding its negative effects, and practicing person-centred communication strategies. | 1. Elderspeak declined from 34.6% (SD = 18.7) at baseline to 21.0% (SD = 20.0) post-intervention and 22.4% (SD = 22.0) at 3-month follow-up. Linear mixed modelling indicated that changes in elderspeak were predicted by the intervention (b = -12.20, p = .028) and baseline elderspeak (b = -0.65, p < .001). 2. RTC declined from 35.7% (SD = 23.2) at baseline to 20.4% (SD = 32.4) post-intervention and 22.3% (SD = 33.7) at 3-month follow-up. Changes in RTC were predicted by changes in elderspeak (b = 0.43, p < .001) and baseline RTC (b = -0.58, p < .001), as well as communication disability (b = 6.05, p = .03) and comorbidity (b = 1.80, p = .002). |
| **Wilson et al. (2012)** | **Formal Caregivers' Perceptions of Effective Communication Strategies while Assisting Residents with Alzheimer's Disease During Activities of Daily Living** | Canada, Care home | To describe caregivers' perceptions of communication strategies that are effective when assisting individuals with Alzheimer's disease (AD) during activities of daily living (ADLs). | Qualitative (semi-structured focus group interviews) | N/A | N/A | Age: Mean 44.6 years (30-62) Gender: female (9), male (1) Roles: Personal Support Workers (PSWs) (9), Registered Nurse (RN) (1) Experience: Mean 12.51 years (2-25 years) Education: Mean 14.7 years (12-18 years) | 10 formal caregivers | Communication strategies defined by Multidimensional Observation Coding Scheme (MOCS)  A. Task-Focused Communication Strategies: 1. Verbal strategies: - One proposition: Presenting a single, clear statement or question to the resident to simplify communication. - Verbatim repetition: Repeating the exact same words to reinforce understanding. - Paraphrased repetition: Repeating the message with different words to aid comprehension. - Introduce task: Informing the resident about the task at hand to prepare them mentally. - Explanation of actions: Describing each step of the care process to ensure residents understand what is happening, helping to reduce anxiety and resistance. - Use of resident’s name: Using the resident’s name to gain their attention and engage them in the activity. - Negotiation: Reaching agreements with residents to encourage cooperation, such as offering a preferred activity after completing a task. - Encouraging comments: Providing positive feedback to motivate the resident. - Multiple verbal strategies: Combining various verbal strategies to enhance communication effectiveness. 2. Non-verbal strategies: - Hand object to the resident: Providing tactile prompts. - Guided touch: Physically guiding the resident's movements to assist them in performing tasks. - Comfort touch: Using touch to provide comfort and reassurance. - Attention touch: Touching the resident to capture their attention and engage them. - Demonstration gesture: Showing residents how to perform an action through gestures. - Pointing: Directing the resident’s attention to specific objects or areas using pointing. - Verbal and visual strategies: Combining verbal instructions with visual cues to aid understanding.  B. Social Communication Strategies: 1. Greeting the resident: establishing initial contact 2. Complimenting the resident: building rapport 3. Responding to the resident: showing empathy and understanding  C. Miscellaneous Communication Strategies: 1. Full physical assistance: Providing complete physical help when necessary. 2. Redirect resident: Redirecting the resident's attention to another activity or topic to manage behaviour and focus.  Emergent Themes 1. General communication strategies - Be patient: Provide time for the resident to respond to a request, instruction, or general communication attempt. - Focus the resident: Gain the resident’s attention and use strategies (e.g., proximity) to help maintain their focus during the activity. - Environmental cues: Use stimulation available in the resident’s environment as cues to support participation in the activity. (e.g. put the light on in the morning) - Eye-contact: Establish eye-contact to introduce yourself to the resident, to connect with the resident, and to gain their attention. - Para-verbal monitoring: Monitor the tone, pitch, and pace of the voice when communicating with the resident. - Interpret non-verbal communication: Be aware of a resident’s use of non-verbal communication. 2. General Care Strategies - Familiarity: Knowing their personal preferences and personal history assists in meeting the resident’s needs and interpreting their behaviour. - Interpret behaviour: Be aware of a resident’s actions to verify that they are compliant and/or understand. - Assess mood: At the beginning of each encounter, assess the resident’s mood and decide the best action to take following their response. - Assess for restiveness: At the beginning of each encounter, assess for any restive behavior and consider the management of aggressive responses. - Request assistance: Ask for assistance from another caregiver because the resident may respond to a different person. - Postpone/repeated attempts: When communication or non-compliance difficulties arise, postpone completing the task and repeat the attempt to complete the task at a later time. | 1. Communication strategies defined by MOCS: - Over three-quarters of the segments derived from the FGI narratives contained at least one strategy identified a priori in MOCS, with verbal task-focused communication strategies being the most common.  - Group consensus during the FGIs identified six communication strategies as useful: negotiation, using the resident’s name, paraphrased repetition, verbatim repetition, greeting the resident, and responding with empathy.  2. Emergent themes: - Caregivers identified 12 strategies to assist individuals with moderate to severe AD during ADLs, classified into two themes: general communication and general care.  3. Differences in strategies used based on disease severity: - Moderate AD: negotiation, giving one instruction at a time, and using familiar objects were more effective. - Severe AD: postponement of the task, interpreting non-verbal behaviour, using the resident's name, and employing full assistance were preferred.  4. Ratings of effectiveness: - Verbal strategies were rated higher than non-verbal strategies. - Encouraging comments, using the resident’s name, and giving one instruction at a time were highly rated. |

## **Appendix 3. Quality Assessment of Included Studies**

Table C.1 Quality Assessment of Qualitative Studies[66]

| **No** | **Studies** | **1. Is there congruity between the stated philosophical perspective and the research methodology?** | **2. Is there congruity between the research methodology and the research question or objectives?** | **3. Is there congruity between the research methodology and the methods used to collect data?** | **4. Is there congruity between the research methodology and the representation and analysis of data?** | **5. Is there congruity between the research methodology and the interpretation of results?** | **6. Is there a statement locating the researcher culturally or theoretically?** | **7. Is the influence of the researcher on the research, and vice- versa, addressed?** | **8. Are participants, and their voices, adequately represented?** | **9. Is the research ethical according to current criteria or, for recent studies, and is there evidence of ethical approval by an appropriate body?** | **10. Do the conclusions drawn in the research report flow from the analysis, or interpretation, of the data?** |
| --- | --- | --- | --- | --- | --- | --- | --- | --- | --- | --- | --- |
| 1 | Allwood et al. (2017) | **Yes** | **Yes** | **Yes** | **Yes** | **Yes** | **No** | **No** | **Yes** | **Yes** | **Yes** |
| 2 | Anantapong et al. (2022) | **Yes** | **Yes** | **Yes** | **Yes** | **Yes** | **No** | **No** | **Yes** | **Yes** | **Yes** |
| 3 | Baillie et al. (2012) | **Yes** | **Yes** | **Yes** | **Yes** | **Yes** | **No** | **No** | **Yes** | **Yes** | **Yes** |
| 4 | Benbow et al. (2011) | **Yes** | **Yes** | **Yes** | **Yes** | **Yes** | **No** | **No** | **Yes** | **Yes** | **Yes** |
| 5 | Kamalraj et al. (2021) | **Yes** | **Yes** | **Yes** | **Yes** | **Yes** | **No** | **No** | **Yes** | **Yes** | **Yes** |
| 6 | Palmer (2012) | **Yes** | **Yes** | **Yes** | **Yes** | **Yes** | **No** | **No** | **Yes** | **No** | **Yes** |
| 7 | Riachi (2017) | **Yes** | **Yes** | **Yes** | **Yes** | **Yes** | **No** | **No** | **Yes** | **Yes** | **Yes** |
| 8 | Saini et al. (2016) | **Yes** | **Yes** | **Yes** | **Yes** | **Yes** | **No** | **No** | **Yes** | **Yes** | **Yes** |
| 9 | Wang et al. (2013) | **Yes** | **Yes** | **Yes** | **Yes** | **Yes** | **No** | **Yes** | **Yes** | **Yes** | **Yes** |
| 10 | Ward et al. (2008) | **Yes** | **Yes** | **Yes** | **Yes** | **Yes** | **No** | **No** | **Yes** | **Yes** | **Yes** |
| 11 | Wheeler and Oyebode (2010) | **Yes** | **Yes** | **Yes** | **Yes** | **Yes** | **No** | **No** | **Yes** | **Yes** | **Yes** |
| 12 | Wilson et al. (2012) | **Yes** | **Yes** | **Yes** | **Yes** | **Yes** | **No** | **No** | **Yes** | **Yes** | **Yes** |

Table C.2 Quality Assessment of Quasi-Experimental Studies[67]

| **No** | **Studies** | **1. Is it clear in the study what is the “cause” and what is the “effect” (i.e. there is no confusion about which variable comes first)?** | **2. Was there a control group?** | **3. Were participants included in any comparisons similar?** | **4. Were the participants included in any comparisons receiving similar treatment/care, other than the exposure or intervention of interest?** | **5. Were there multiple measurements of the outcome, both pre and post the intervention/exposure?** | **6. Were the outcomes of participants included in any comparisons measured in the same way?** | **7. Were outcomes measured in a reliable way?** | **8. Was follow-up complete and if not, were differences between groups in terms of their follow-up adequately described and analysed?** | **9. Was appropriate statistical analysis used?** |
| --- | --- | --- | --- | --- | --- | --- | --- | --- | --- | --- |
| 1 | Acton et al. (2007) | **Yes** | **No** | **Yes** | **No** | **Yes** | **Yes** | **Yes** | **Unclear** | **Yes** |
| 2 | Alnes et al. (2011) | **Yes** | **Yes** | **Yes** | **Yes** | **Yes** | **Yes** | **Yes** | **Unclear** | **Unclear** |
| 3 | Barbosa et al. (2016) | **Yes** | **Yes** | **Unclear** | **Yes** | **Yes** | **Yes** | **Yes** | **Yes** | **Yes** |
| 4 | Barbosa et al. (2016) | **Yes** | **Yes** | **Unclear** | **Yes** | **Yes** | **Yes** | **Yes** | **Yes** | **Yes** |
| 5 | Bourgeois et al. (2004) | **Yes** | **Yes** | **Yes** | **Yes** | **Yes** | **Yes** | **Yes** | **Yes** | **Yes** |
| 6 | Burshnic and Bourgeois (2022) | **Yes** | **No** | **Yes** | **Yes** | **Yes** | **Yes** | **Yes** | **Yes** | **Yes** |
| 7 | Douglas and MacPherson (2021) | **Yes** | **No** | **Yes** | **Yes** | **Yes** | **Yes** | **Yes** | **Yes** | **Yes** |
| 8 | Kaasalainen et al. (2021) | **Yes** | **No** | **Yes** | **Yes** | **Yes** | **Yes** | **Yes** | **No** | **Yes** |
| 9 | Passalacqua and Harwood (2012) | **Yes** | **No** | **Yes** | **Yes** | **Yes** | **Yes** | **No** | **Yes** | **Yes** |
| 10 | Sánchez-Martínez et al. (2023) | **Yes** | **No** | **Yes** | **Yes** | **Yes** | **Yes** | **Yes** | **Yes** | Not Applicable |

Table C.3 Quality Assessment of Cross-Sectional Studies [68]

| **No** | **Studies** | **1. Were the criteria for inclusion in the sample clearly defined?** | **2. Were the study subjects and the setting described in detail?** | **3. Was the exposure measured in a valid and reliable way?** | **4. Were objective, standard criteria used for measurement of the condition?** | **5. Were confounding factors identified?** | **6. Were strategies to deal with confounding factors stated?** | **7. Were the outcomes measured in a valid and reliable way?** | **8. Was appropriate statistical analysis used?** |
| --- | --- | --- | --- | --- | --- | --- | --- | --- | --- |
| 1 | Dooley et al. (2018) | **Yes** | **Yes** | **Yes** | **Yes** | **Unclear** | **No** | **Yes** | **Yes** |
| 2 | Mundadan et al. (2023) | **Yes** | **Yes** | **Yes** | **Yes** | **No** | **No** | **Yes** | **Unclear** |
| 3 | Savundranayagam et al. (2014) | **No** | **No** | **Yes** | **Yes** | **No** | **No** | **Yes** | **Unclear** |
| 4 | Savundranayagam and Moore-Nielsen (2015) | **No** | **No** | **Yes** | **Yes** | **No** | **No** | **Yes** | **Yes** |
| 5 | Savundranayagam et al. (2016) | **No** | **No** | **Yes** | **Yes** | **No** | **No** | **Yes** | **Yes** |
| 6 | Shaw et al. (2022) | **Yes** | **Yes** | **Yes** | **Yes** | **Yes** | **No** | **Yes** | **Yes** |
| 7 | Williams et al. (2009) | **Yes** | **Yes** | **Yes** | **Yes** | **Unclear** | **No** | **Yes** | **Yes** |

Table C.4 Quality Assessment of Randomised Controlled Trials [69]

| **No** | **Studies** | **1. Was true randomization used for assignment of participants to treatment groups?** | **2. Was allocation to treatment groups concealed?** | **3. Were treatment groups similar at the baseline?** | **4. Were participants blind to treatment assignment?** | **5. Were those delivering the treatment blind to treatment assignment?** | **6. Were treatment groups treated identically other than the intervention of interest?** | **7. Were outcome assessors blind to treatment assignment?** | **8. Were outcomes measured in the same way for treatment groups?** | **9. Were outcomes measured in a reliable way** | **10. Was follow up complete and if not, were differences between groups in terms of their follow up adequately described and analysed?** | **11. Were participants analysed in the groups to which they were randomized?** | **12. Was appropriate statistical analysis used?** | **13. Was the trial design appropriate and any deviations from the standard RCT design (individual randomization, parallel groups) accounted for in the conduct and analysis of the trial?** |
| --- | --- | --- | --- | --- | --- | --- | --- | --- | --- | --- | --- | --- | --- | --- |
| 1 | Bourgeois et al. (2001) | **Unclear** | **Unclear** | **Yes** | **No** | **No** | **Yes** | **No** | **Yes** | **Yes** | **Yes** | **Yes** | **Yes** | **No** |
| 2 | Williams et al. (2017) | **Yes** | **Unclear** | **Yes** | **No** | **No** | **Yes** | **Yes** | **Yes** | **Yes** | **Yes** | **Yes** | **Yes** | **Yes** |

Table C.5 Quality Assessment of Mixed Method Studies [26]

| **Category of study designs** | **Methodological quality criteria** | **Studies** | |
| --- | --- | --- | --- |
|  |  | **Berry et al. (2023)** | **Song et al. (2019)** |
| Screening questions (for all types) | S1. Are there clear research question | Yes | Yes |
|  | S2. Do the collected data allow to address the research questions? | Yes | Yes |
| 1. Qualitative | 1.1. Is the qualitative approach appropriate to answer the research question? | Yes | Yes |
|  | 1.2. Are the qualitative data collection methods adequate to address the research question? | Yes | Yes |
|  | 1.3. Are the findings adequately derived from the data? | Yes | Yes |
|  | 1.4. Is the interpretation of results sufficiently substantiated by data? | Yes | No |
|  | 1.5. Is there coherence between qualitative data sources, collection, analysis and interpretation? | Yes | Unclear |
| 2. Quantitative randomized controlled trials | 2.1. Is randomization appropriately performed? |  | Yes |
|  | 2.2. Are the groups comparable at baseline? |  | Yes |
|  | 2.3. Are there complete outcome data? |  | Yes |
|  | 2.4. Are outcome assessors blinded to the intervention provided? |  | Unclear |
|  | 2.5 Did the participants adhere to the assigned intervention? |  |  |
| 3. Quantitative nonrandomized | 3.1. Are the participants representative of the target population? |  |  |
|  | 3.2. Are measurements appropriate regarding both the outcome and intervention (or exposure)? |  |  |
|  | 3.3. Are there complete outcome data? |  |  |
|  | 3.4. Are the confounders accounted for in the design and analysis? |  |  |
|  | 3.5. During the study period, is the intervention administered (or exposure occurred) as intended? |  |  |
| 4. Quantitative descriptive | 4.1. Is the sampling strategy relevant to address the research question? | Yes |  |
|  | 4.2. Is the sample representative of the target population? | Unclear |  |
|  | 4.3. Are the measurements appropriate? | Yes |  |
|  | 4.4. Is the risk of nonresponse bias low? | Yes |  |
|  | 4.5. Is the statistical analysis appropriate to answer the research question? | Yes |  |
| 5. Mixed methods | 5.1. Is there an adequate rationale for using a mixed methods design to address the research question? | Yes | Yes |
|  | 5.2. Are the different components of the study effectively integrated to answer the research question? | Yes | Yes |
|  | 5.3. Are the outputs of the integration of qualitative and quantitative components adequately interpreted? | Yes | Yes |
|  | 5.4. Are divergences and inconsistencies between quantitative and qualitative results adequately addressed? | Unclear | No |
|  | 5.5. Do the different components of the study adhere to the quality criteria of each tradition of the methods involved? | Yes | Yes |
